# c-Myc-activated USP2-AS1 suppresses senescence and promotes tumor progression via stabilization of E2F1 mRNA

**DOI:** 10.1038/s41419-021-04330-2

**Published:** 2021-10-27

**Authors:** Bingyan Li, Guang Zhang, Zhongyu Wang, Yang Yang, Chenfeng Wang, Debao Fang, Kaiyue Liu, Fang Wang, Yide Mei

**Affiliations:** 1 The First Affiliated Hospital of USTC, Hefei National Laboratory for Physical Sciences at Microscale, School of Basic Medical Sciences, Division of Life Sciences and Medicine, University of Science and Technology of China, Hefei, Anhui China; 2 Biomedical Sciences and Health Laboratory of Anhui Province, University of Science and Technology of China, Hefei, Anhui China

**Keywords:** Oncogenes, Senescence

## Abstract

The c-Myc oncoprotein plays a prominent role in cancer initiation, progression, and maintenance. Long noncoding RNAs (lncRNAs) are recently emerging as critical regulators of the c-Myc signaling pathway. Here, we report the lncRNA USP2-AS1 as a direct transcriptional target of c-Myc. Functionally, USP2-AS1 inhibits cellular senescence and acts as an oncogenic molecule by inducing E2F1 expression. Mechanistically, USP2-AS1 associates with the RNA-binding protein G3BP1 and facilitates the interaction of G3BP1 to E2F1 3′-untranslated region, thereby leading to the stabilization of E2F1 messenger RNA. Furthermore, USP2-AS1 is shown as a mediator of the oncogenic function of c-Myc via the regulation of E2F1. Together, these findings suggest that USP2-AS1 is a negative regulator of cellular senescence and also implicates USP2-AS1 as an important player in mediating c-Myc function.

## Introduction

Cellular senescence is defined as an irreversible cell cycle arrest triggered by diverse forms of stresses such as telomere erosion/uncapping, DNA damage, oxidative stress, and oncogenic activation [1–4]. Senescent cells are characterized by a variety of phenotypes including morphological and metabolic changes, senescence-associated β-galactosidase (SA-β-gal) activity, formation of senescence-associated heterochromatin foci (SAHF), and the senescence-associated secretory phenotype (SASP) [5–8]. The cellular senescence program is principally established and maintained by two interplaying and partially exclusive p53/p21 and p16/Rb tumor-suppressive pathways [9, 10]. p21 and p16 are cyclin-dependent kinase (CDK) inhibitors and potent negative regulators of cell cycle progression. During senescence induction, p21 and p16 are able to prevent CDK-dependent phosphorylation of Rb, thereby leading to Rb-mediated E2F1 inhibition and senescence-associated cell cycle arrest [11–15]. Although the executive pathways of cellular senescence have been extensively studied, the underlying control mechanisms still remain elusive.

The oncoprotein c-Myc plays a pivotal role in cancer initiation, progression, and maintenance [16, 17]. The expression of c-Myc is tightly controlled in normal cells, but becomes dysregulated and overexpressed in more than half of human cancers through chromosomal translocation, gene amplification, or insertional mutagenesis [18, 19]. As a master transcriptional regulator, c-Myc targets 10 to 15% of genes in the human genome [20, 21]. c-Myc is also suggested as a global amplifier of transcription at active loci [22, 23]. In agreement with a large number of c-Myc target genes, overexpression of c-Myc deregulates a series of biological processes such as cell cycle progression, cell growth, cell metabolism, protein synthesis, and genomic maintenance [24–26]. Such deregulation provides growth advantages for tumors and contributes to the oncogenic function of c-Myc. Of interest, the suppression of c-Myc triggers cellular senescence in diverse tumor types, while overexpression of c-Myc inhibits oncogene-induced senescence in melanoma [27, 28]. These findings establish an important role of c-Myc in negatively regulating cellular senescence in established tumors. They also indicate that suppression of cellular senescence is critical for the tumor-promoting effect of c-Myc.

Emerging evidence demonstrates that long noncoding RNAs (lncRNAs) are important regulators of gene expression and influence a variety of cellular processes through multiple diverse mechanisms [29, 30]. The role of lncRNAs in cancer initiation and progression is increasingly recognized [31, 32]. In particular, a growing number of lncRNAs have been shown to regulate the c-Myc oncogenic pathway [33–38]. For instance, the c-Myc-responsive lncRNA IDH1-AS1 functions as a tumor-suppressive molecule via a metabolic mechanism [39]. In addition, we have recently shown that c-Myc transcriptionally activates the expression of the lncRNA E2F1 messenger RNA (mRNA)-stabilizing factor (EMS) that connects c-Myc to cell cycle control and tumorigenesis [40]. However, it remains unclear whether c-Myc could exert its oncogenic role via lncRNAs that have a cellular senescence-regulatory function.

In this study, we show that the lncRNA USP2-AS1 is transcriptionally upregulated by c-Myc. USP2-AS1 inhibits cellular senescence and functions as an oncogenic lncRNA. Mechanistically, USP2-AS1 interacts with the RNA-binding protein G3BP1 to stabilize E2F1 mRNA and increase E2F1 expression. Moreover, USP2-AS1 is able to mediate the oncogenic function of c-Myc via the regulation of E2F1. Our study implicates USP2-AS1 as an important regulator of cellular senescence and provides new insights into the mechanisms of c-Myc oncogenic activity.

## Materials and methods

### Reagents and antibodies

The following reagents and antibodies used in this study were purchased from the indicated sources: actinomycin D (Abcam, ab141058), Lipofectamine 2000 (Invitrogen), M2 beads (Sigma), 4′,6-diamidino-2-phenylindole (Sigma), streptavidin-coated agarose beads (Thermo Fisher Scientific), complete EDTA-free protease inhibitor cocktail (Roche Applied Science), antibodies against E2F1 (Cell Signaling, 3742S, 1:1000), G3BP1 (Proteintech, 13057-2-AP, 1:2000), RARP (Santa Cruz, sc-8007, 1:1000), Flag (Sigma, #F3165, 1:4000), c-Myc for chromatin immunoprecipitation (ChIP) assay (Cell Signaling, 9402S), c-Myc for western blotting (Cell Signaling, 9402S, 1:1000), glyceraldehyde 3-phosphate dehydrogenase (GAPDH) (Santa Cruz, sc-166545, 1:5000), H3K9me3 (Abcam, ab176916, 1:200), horseradish peroxidase-conjugated secondary antibodies against mouse (115-035-062) and rabbit (111-035-144) (Jackson ImmunoResearch, 1:10,000).

### Cell culture

A549 cells were cultured in Roswell Park Memorial Institute (RPMI) 1640 medium (Sigma) supplemented with 10% fetal bovine serum (FBS) and antibiotics. HCT116, IMR90, and HEK293T cell lines were maintained in Dulbecco’s modified Eagle’s medium (DMEM) (Gibco) supplemented with 10% FBS and antibiotics. All cell lines were routinely tested for mycoplasma contamination before they were used for experiments.

### Real-time reverse transcription-polymerase chain reaction (RT-PCR)

Total RNA was isolated using Trizol (Invitrogen). One microgram of RNA was used to synthesize complementary DNA (cDNA) using HiScript® III RT SuperMix (Vazyme, China) according to the manufacturer’s instruction. Real-time PCR was performed using SYBR Green Master Mix (Vazyme, China) and analyzed with the StepOnePlus real-time PCR system (Thermo Fisher Scientific). The PCR results, recorded as threshold cycle numbers (Ct), were normalized against an internal control (GAPDH). The expression data were analyzed using the 2^−ΔΔCT^ method. The primer sequences were shown in Supplementary Table S1.

### Rapid amplification of cDNA ends (RACE)

5′-RACE and 3′-RACE were performed using the SMARTer® 5′/3′ RACE Kit (Clontech) according to the manufacturer’s instructions. The primers P1 and P2 were used for 5′-RACE. The primers P3 and P4 were used for 3′-RACE. P1 and P4 were supplied in the kit. The primer sequences of P2 and P3 were shown in Supplementary Table S1.

### Generation of the lentiviral expression system

To generate lentiviruses expressing the indicated short hairpin RNAs (shRNAs), HEK293T cells were transfected with shRNAs (cloned in PLKO.1), pREV, pGag/Pol/PRE, and pVSVG with a ratio of 2:2:2:1. To generate lentiviruses expressing USP2-AS1 or the indicated proteins, HEK293T cells were transfected with pSin-based construct together with pmd2.g and pspax2 with a ratio of 2:1:2. For the generation of the control virus, PLKO.1 containing control shRNA or pSin empty vector was used. Twelve hours after transfection, cells were cultured in a fresh medium for another 24 h. The culture medium containing lentivirus particles was filtered through a 0.45-μm PVDF filter and used for infection. Twenty-four hours after infection, target cells were subjected to puromycin (2 μg/ml) selection for another 24 h before they were used for the functional experiments. The successful knockdown and overexpression were verified by real-time RT-PCR and western blot analysis in each experiment. The shRNA target sequences are listed in Supplementary Table S1.

### Plasmid construction

All the plasmids used in this study were constructed by standard molecular biology techniques. The coding sequences of c-Myc, E2F1, and G3BP1, cDNA of full-length USP2-AS1, and cDNA of full-length E2F1 3′-untranslated region (3′-UTR) were obtained by RT-PCR. These DNA fragments were then cloned into the lentiviral expression vector pSin-EF2 or the luciferase reporter vector psi-CHECK2. The shRNA-resistant USP2-AS1 was generated by PCR-based mutagenesis and cloned into pSin-EF2 vector. All the plasmids were confirmed by sequencing. For both generation of lentiviruses and transient overexpression experiments, pSin-based constructs were used.

### RNA immunoprecipitation (RIP)

RIP was performed as we previously described [40]. A549 cells expressing control or Flag-G3BP1 were lysed in IP lysis buffer (50 mM Tris-HCl, pH 7.4, 150 mM NaCl, 1.5 mM MgCl_2_, 1 mM EDTA, 0.5% NP-40) supplemented with 1× protease inhibitor cocktail, RNase A inhibitor, and DNase I. Cell lysates were incubated with M2 beads at 4 °C for 6 h. After extensive washing, the beads-bound immunocomplexes were eluted using elution buffer (50 mM Tris-HCl, pH 8.0, 1% sodium dodecyl sulfate (SDS), and 10 mM EDTA) at 65 °C for 10 min. RNAs and proteins in the eluents were then subjected to real-time RT-PCR and western blot analyses, respectively.

### In vitro transcription of USP2-AS1 and its antisense RNA

To synthesize USP2-AS1 and its antisense RNA, the DNA template used in the transcription system was generated by RT-PCR using forward and reverse primers containing the T7 RNA polymerase promoter sequence, respectively. PCR products were purified using DNA Gel Extraction Kit (AxyPrep). In vitro transcription was then performed using MaxiScript T7 Kit (Ambion) according to the manufacturer’s instructions. To verify the interaction of USP2-AS1 with G3BP1, in vitro synthesized USP2-AS1 or its antisense RNA was incubated with purified recombinant Flag-G3BP1 bound with M2 beads. After incubation and extensive washing, the beads-bound RNAs were eluted as templates for RT-PCR analysis.

### Biotin pull-down assay

All the process was performed in the RNase-free condition. Sense or antisense biotin-labeled DNA oligomers corresponding to USP2-AS1 were incubated with lysates from 5 × 10^6^ A549 cells. One hour after incubation, streptavidin-coated beads (Thermo Fisher Scientific) were added to isolate the RNA–protein complex, followed by western blot and real-time RT-PCR analyses.

### ChIP assay

The ChIP assay was performed as we previously described [41]. Briefly, A549 cells were cross-linked with 1% formaldehyde for 10 min. The ChIP assay was then performed using the ChIP Assay Kit (Beyotime, Shanghai, China) with anti-c-Myc antibody or normal rabbit immunoglobulin G (IgG) according to the manufacturer’s protocol. The bound DNA fragments were subjected to real-time PCR analysis using the specific primers (Supplementary Table S1).

### Luciferase reporter assay

To investigate whether USP2-AS1 is transcriptionally regulated by c-Myc, A549 cells expressing control shRNA or c-Myc shRNA, or A549 cells expressing control or Flag-c-Myc proteins were transfected with pGL3, pGL3-BS1, or pGL3-BS1 Mut construct plus Renilla luciferase reporter plasmid as indicated. To examine the effect of USP2-AS1 on E2F1 3′-UTR, A549 cells transduced with the indicated lentiviruses were transfected with psi-CHECK2-E2F1 3′-UTR. Twenty-four hours after transfection, firefly and Renilla luciferase activities were measured by the Dual-Luciferase Reporter Assay System (Promega), and Renilla activity was used to normalize firefly activity.

### RNA-sequencing (RNA-seq) and analysis

To identify genes that are regulated by USP2-AS1, A549 cells were infected with lentiviruses expressing control shRNA or USP2-AS1 shRNA. Seventy-two hours after infection, total RNA was extracted for RNA-seq. RNA-seq was performed and analyzed by Novogene (Beijing, China) (two biological replicates per group). Raw read counts were used for differential gene expression analysis by DESeq2. Genes with adjusted *p* value <0.05 and log 2 FC (fold change) ≥1.0 were counted as differentially expressed genes. The sequencing data have been deposited in the National Center for Biotechnology Information Gene Expression Omnibus with accession code GSE169138.

To compare the expression levels of different USP2-AS1 transcripts in cells, we utilized our previously published RNA-seq data (SRP171977) [40]. These data were analyzed by Cutadapt v1.18 to remove adapters and low-quality reads. Clean reads were then aligned to human reference genome assembly version GRCh38/hg38 using STAR_2.6.1a and assembled by StringTie. All of the assemblies and the reference transcriptome annotation were merged by StringTie-merge. All of the aligned bam files were visualized in IGV. StringTie-e was used to calculate transcripts per million of different USP2-AS1 transcripts.

### Colony formation assay

For colony formation assay, 1 × 10^3^ HCT116 or A549 cells were seeded in a 6-well plate. For HCT116 cells, colonies formed on the plate were stained with crystal violet after 8 days of incubation. For A549 cells, colonies formed on the plate were stained with crystal violet after 12 days of incubation. The number of all colonies on the plate was then counted. Data shown are mean ± SD from three independent biological replicates.

### EdU incorporation assay

The EdU incorporation assay was performed with an EdU Assay Kit (Guangzhou RiboBio, Guangzhou, China) according to the manufacturer’s instructions. Briefly, cells were incubated with DMEM medium containing 50 μM EdU for 2 h. The nuclei were also stained with Hoechst 33342 (Sigma), and the images were acquired with an Olympus DP73 microscope (Olympus).

### Cell senescence assay

Senescence assay was conducted using the Senescence Detection Kit from Beyotime (Shanghai, China). Briefly, HCT116 or A549 cells were infected with the indicated lentiviruses. Ninety-six hours post infection, cells were fixed by fixative solution for 20 min at room temperature. After washing twice with phosphate-buffered saline, cells were stained with 0.1% X-gal solution for 48 h at 37 °C. The X-gal stained cells were counted under a microscope.

### Xenograft mouse model

For xenograft experiments, 2 × 10^6^ HCT116 cells were injected into the left flank or right flank of 5-week-old male athymic nude mice (Shanghai SLAC Laboratory Animal Co. Ltd) (*n* = 6 for each group). Mice were used in the experiment at random. Six days after injection, tumor volumes were measured every 6 days with a caliper and calculated using the equation: volume = length × width^2^ × 0.52. Twenty-four days after injection, mice were sacrificed and tumors were excised and weighed. During testing the tumors’ weight, the experimentalists were blinded to the information of tumor tissues. The extracted RNAs and proteins from the excised tumors were subjected to real-time RT-PCR and western blot analyses, respectively.

### Ethics statement

Studies on mice were conducted in accordance with relevant guidelines and regulations and were approved by the Animal Research Ethics Committee of the University of Science and Technology of China (ethics number 202012280951000537337).

### Statistical analysis

Statistical analysis was carried out using Microsoft Excel software and GraphPad Prism to assess differences between experimental groups. Statistical significance was analyzed by Student’s *t* test and expressed as a *p* value. *P* values <0.05 were considered to be statistically significant. One asterisk, two asterisks, and three asterisks indicate *p* < 0.05, *p* < 0.01, and *p* < 0.001, respectively; n.s. indicates no significance.

## Results

### USP2-AS1 suppresses cellular senescence and functions as an oncogenic lncRNA

To generate new insights into the molecular mechanisms whereby c-Myc promotes tumorigenesis, we sought to identify c-Myc-responsive lncRNAs with regulatory functions in cellular senescence. By analyzing our recently published RNA-seq data generated in A549 cells with or without c-Myc induction and public ENCODE c-Myc ChIP-seq datasets [40, 42], six indicated lncRNAs with no or little functional characterization were revealed as potential transcriptional targets of c-Myc (Supplementary Fig. S1A). Five c-Myc-upregulated lncRNAs were further validated by real-time RT-PCR analysis in A549 cells (Supplementary Fig. S1B). Functionally, knockdown of USP2-AS1, but not other lncRNAs, dramatically increased cellular senescence, as manifested by enhanced SA-β-gal activity in USP2-AS1 knockdown A549 cells (Supplementary Fig. S1C, D), indicating the specific inhibitory effect of USP2-AS1 on cellular senescence.

According to Ensemble annotation, multiple transcripts could be transcribed from the *USP2-AS1* gene. By analyzing the above-mentioned RNA-seq data [43], ENST00000498979 was found to be the most abundantly expressed USP2-AS1 transcript in both control and c-Myc-overexpressed A549 cells (Supplementary Fig. S1E, F). We, therefore, focused on this USP2-AS1 transcript in our study. By performing 5′- and 3′-RACE experiments, USP2-AS1 (ENST00000498979) was revealed as an RNA transcript with a molecular size of 2486 nt (Supplementary Fig. S1G, H and Supplementary Tables S2 and 3). This USP2-AS1 transcript was predominantly localized in the cytoplasm (Supplementary Fig. S1I, J).

**Fig. 1:**
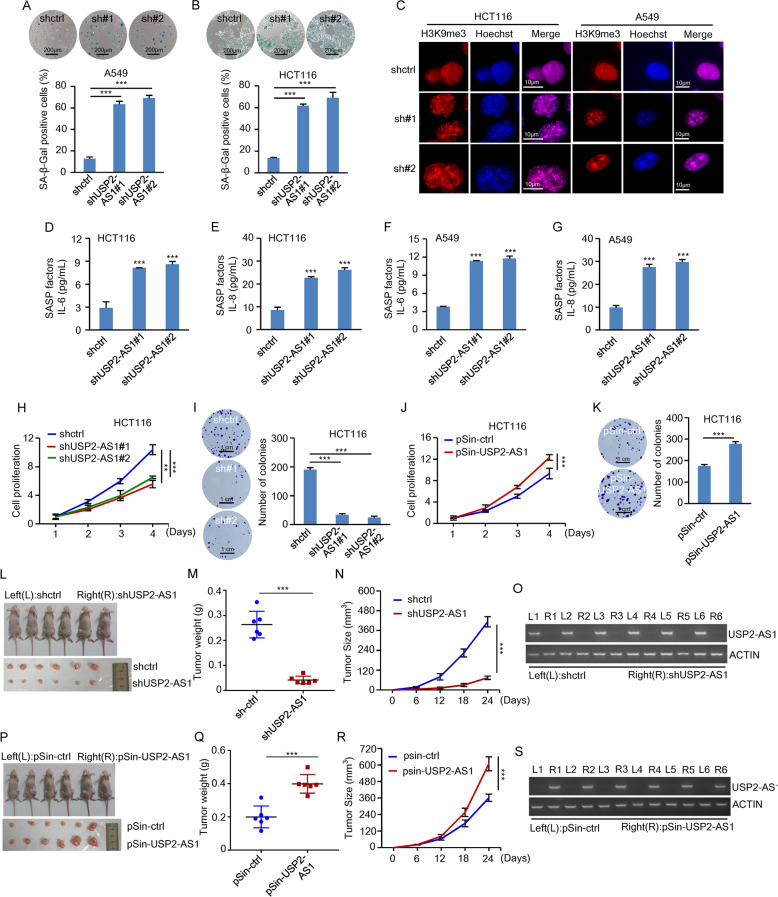
USP2-AS1 suppresses cellular senescence and acts as an oncogenic lncRNA. **A**, **B** A549 (**A**) and HCT116 (**B**) cells were infected with lentiviruses expressing control shRNA, USP2-AS1 shRNA#1, or USP2-AS1 shRNA#2. Ninety-six hours later, cells were subjected to β-galactosidase staining. The shown images are representative of three independent experiments. Data shown are mean ± SD (*n* = 3). ***P < 0.001. The knockdown efficiency of USP2-AS1 in these cells was shown in Supplementary Fig. S2C, D. **C** A549 and HCT116 cells were infected with lentiviruses expressing control shRNA, USP2-AS1 shRNA#1, or USP2-AS1 shRNA#2. Ninety-six hours later, cells were immunostained with anti-H3K9me3 antibody to examine senescence-associated heterochromatin foci (SAHF) formation. The nuclei were also visualized by DAPI staining. The number of SAHF per cell was counted and shown in Supplementary Fig. S2A, B. **D**–**G** A549 and HCT116 cells were infected with lentiviruses expressing control shRNA, USP2-AS1 shRNA#1, or USP2-AS1 shRNA#2. Ninety-six hours later, the extracellular levels of IL-6 and IL-8 were examined by using ELISA assay. Data shown are mean ± SD (*n* = 3). ***P < 0.001. **H** Shown are the growth curves of HCT116 cells expressing control shRNA, USP2-AS1 shRNA#1, or USP2-AS1 shRNA#2. Data shown are mean ± SD (*n* = 3). **P < 0.01 and ***p < 0.001. **I** Colonies of HCT116 cells expressing control shRNA, USP2-AS1 shRNA#1, or USP2-AS1 shRNA#2 were stained with crystal violet after 8 days of incubation. The shown images are representative of three independent experiments. Data shown are mean ± SD (*n* = 3). ***P < 0.001. **J** Shown are the growth curves of HCT116 cells expressing control or USP2-AS1. Data shown are mean ± SD (*n* = 3). ***P < 0.001. The successful overexpression of USP2-AS1 was shown in Supplementary Fig. S2K. **K** Colonies of HCT116 cells expressing control or USP2-AS1 were stained with crystal violet after 8 days of incubation. The shown images are representative of three independent experiments. Data shown are mean ± SD (*n* = 3). ***P < 0.001. **L**–**O** A total of 2 × 10^6^ HCT116 cells expressing either control shRNA or USP2-AS1 shRNA were individually injected to the left and right flanks of nude mice as indicated (*n* = 6 for each group). Representative photographs of mice and xenograft tumors were taken 24 days after injection (**L**). The excised tumors were weighed (**M**). Tumor sizes were measured at the indicated time points (**N**). RNA from the excised xenografts was also analyzed by both RT-PCR (**O**) and real-time RT-PCR (Supplementary Fig. S2O). ***P < 0.001. **P**–**S** A total of 2 × 10^6^ HCT116 cells expressing either control or USP2-AS1 were individually injected to the left and right flanks of nude mice as indicated (*n* = 6 for each group). Representative photographs of mice and xenograft tumors were taken 24 days after injection (**P**). The excised tumors were weighed (**Q**). Tumor sizes were measured at the indicated time points (**R**). RNA from the excised xenografts was also analyzed by both RT-PCR (**S**) and real-time RT-PCR (Supplementary Fig. S2P). ***P < 0.001.

To further confirm the regulatory function of USP2-AS1 in cellular senescence, we used an additional shRNA to knockdown USP2-AS1. Knockdown of USP2-AS1 consistently and strongly increased SA-β-gal activity, SAHF formation, and enhanced the SASP as represented by elevated extracellular levels of interleukin-6 (IL-6) and IL-8 in both HCT116 and A549 cells (Fig. 1A–G and Supplementary Fig. S2A–D). This USP2-AS1 knockdown-increased SA-β-gal activity could be reversed by the expression of exogenous USP2-AS1 (Supplementary Fig. S2E–H). These data suggest that USP2-AS1 indeed suppresses cellular senescence.

Given the ability of USP2-AS1 to suppress cellular senescence, we next investigated whether USP2-AS1 could act as an oncogenic molecule. Knockdown of USP2-AS1 in both HCT116 and A549 cells greatly decreased the proliferation and number of colonies (Fig. 1H, I and Supplementary Fig. S2I, J). In contrast, ectopic expression of USP2-AS1 in these cells led to an increase in the proliferation and colony number (Fig. 1J, K and Supplementary Fig. S2K–N). By using a xenograft mouse model, USP2-AS1 knockdown was shown to significantly inhibit in vivo xenograft tumor growth of HCT116 cells (Fig. 1L–O and Supplementary Fig. S2O). Conversely, USP2-AS1 overexpression in HCT116 cells markedly promoted in vivo xenograft tumor growth (Fig. 1P–S and Supplementary Fig. S2P). Taken together, these findings suggest that USP2-AS1 functions as an oncogenic lncRNA.

### USP2-AS1 is transcriptionally upregulated by c-Myc

**Fig. 2:**
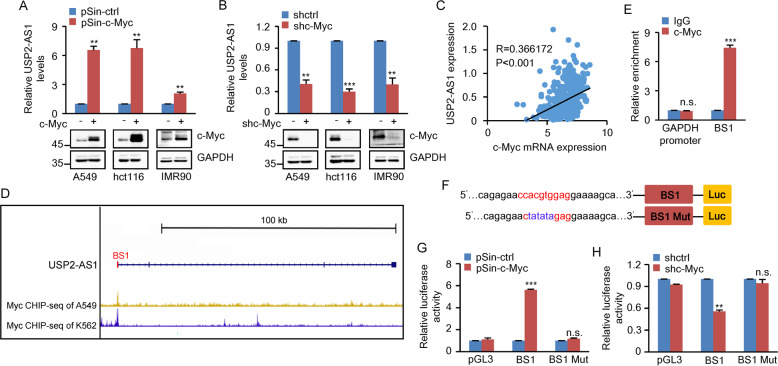
USP2-AS1 is a direct transcriptional target of c-Myc. **A** The indicated cells were infected with lentiviruses expressing control or c-Myc. Forty-eight hours later, total RNA was analyzed by real-time RT-PCR. Data shown are mean ± SD (*n* = 3). **P < 0.01. **B** The indicated cells were infected with lentiviruses expressing control shRNA or c-Myc shRNA. Forty-eight hours later, total RNA was analyzed by real-time RT-PCR. Data shown are mean ± SD (*n* = 3). **P < 0.01 and ***p < 0.001. **C** The expression levels of c-Myc and USP2-AS1 are positively correlated in TCGA colon adenocarcinoma (COAD). **D** Encode ChIP-seq data for c-Myc are displayed in the UCSC browser illustrations. BS1 represents a putative c-Myc-binding site predicted by the JASPAR database. **E** Lysates from A549 cells were subjected to ChIP assay using anti-c-Myc antibody or an isotype-matched control IgG. ChIP products were amplified by real-time RT-PCR. Data shown are mean ± SD (*n* = 3). ***P < 0.001; n.s., no significance. **F** Shown are the pGL3-based wild-type and mutant reporter constructs used for luciferase assay. **G** A549 cells transduced with lentiviruses expressing control or c-Myc were co-transfected with the indicated pGL3-based reporter constructs plus Renilla luciferase plasmid. Twenty-four hours after transfection, reporter activity was measured and plotted after normalizing with respect to Renilla luciferase activity. Data shown are mean ± SD (*n* = 3). ***P < 0.001; n.s., no significance. **H** A549 cells expressing control shRNA or c-Myc shRNA were co-transfected with the indicated reporter constructs. Reporter activity was then measured and plotted after normalizing with respect to Renilla luciferase activity. Data shown are mean ± SD (*n* = 3). **P < 0.01; n.s., no significance.

As mentioned above, USP2-AS1 was identified as a c-Myc-upregulated lncRNA in A549 cells (Supplementary Fig. S1B). We, therefore, sought to further verify the effect of c-Myc on USP2-AS1 expression in more cell lines. Induction of c-Myc increased, whereas knockdown of c-Myc decreased, USP2-AS1 expression in different cell lines including A549, HCT116, and IMR90 (Fig. 2A, B), corroborating the positive effect of c-Myc on USP2-AS1 cellular expression. In support, analysis of the TCGA database showed that the expression levels of c-Myc and USP2-AS1 are indeed positively correlated in different types of cancers, including colon adenocarcinoma (COAD), rectum adenocarcinoma (READ), breast-invasive carcinoma (BRCA), prostate adenocarcinoma (PRAD), and stomach adenocarcinoma (STAD) (Fig. 2C and Supplementary Fig. S3A–D).

To explore how USP2-AS1 is regulated by c-Myc, we first analyzed ENCODE c-Myc ChIP-seq datasets. The results showed the enrichment of c-Myc in the first exon region of the *USP2-AS1* gene (Fig. 2D). After inspection of this region using the JASPAR database, one putative c-Myc-binding site (BS1) was identified (Fig. 2D). The ChIP assay verified the interaction of c-Myc with the chromatin fragment comprising the BS1 site (Fig. 2E). To further determine whether the BS1 site confers c-Myc-dependent transcriptional activity, a luciferase reporter assay was performed. The transcriptional activity of luciferase reporter containing the wild-type BS1 site, but not the mutant BS1 site, was increased by c-Myc overexpression and decreased by c-Myc knockdown (Fig. 2F–H). Taken together, these data indicate that USP2-AS1 is a direct transcriptional target of c-Myc.

### USP2-AS1 exerts its oncogenic role by increasing E2F1 expression

**Fig. 3:**
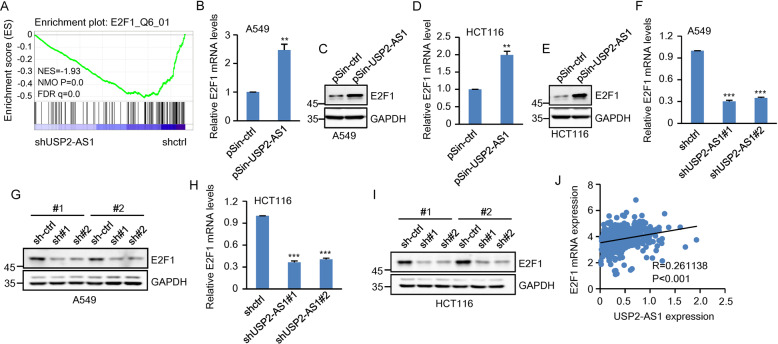
USP2-AS1 increases E2F1 expression. **A** Gene set enrichment analysis (GSEA) revealed downregulation of E2F1 target genes in USP2-AS1 knockdown A549 cells. **B** Total RNA from A549 cells transduced with lentiviruses expressing control or USP2-AS1 was analyzed by real-time RT-PCR. Data shown are mean ± SD (*n* = 3). **P < 0.01. The successful overexpression of USP2-AS1 was shown in Supplementary Fig. S4B. **C** Lysates from A549 cells transduced with lentiviruses expressing control or USP2-AS1 were analyzed by western blotting. **D** Total RNA from HCT116 cells transduced with lentiviruses expressing control or USP2-AS1 was analyzed by real-time RT-PCR. Data shown are mean ± SD (*n* = 3). **P < 0.01. The successful overexpression of USP2-AS1 was shown in Supplementary Fig. S4C. **E** Lysates from HCT116 cells transduced with lentiviruses expressing control or USP2-AS1 were analyzed by western blotting. **F** Total RNA from A549 cells expressing control shRNA, USP2-AS1 shRNA#1, or USP2-AS1 shRNA#2 was analyzed by real-time RT-PCR. Data shown are mean ± SD (*n* = 3). ***P < 0.001. The knockdown efficiency of USP2-AS1 was shown in Supplementary Fig. S4D. **G** Lysates from A549 cells expressing control shRNA, USP2-AS1 shRNA#1, or USP2-AS1 shRNA#2 were analyzed by western blotting. **H** Total RNA from HCT116 cells expressing control shRNA, USP2-AS1 shRNA#1, or USP2-AS1 shRNA#2 was analyzed by real-time RT-PCR. Data shown are mean ± SD (*n* = 3). ***P < 0.001. The knockdown efficiency of USP2-AS1 was shown in Supplementary Fig. S4E. **I** Lysates from HCT116 cells expressing control shRNA, USP2-AS1 shRNA#1, or USP2-AS1 shRNA#2 were analyzed by western blotting. **J** The expression levels of USP2-AS1 and E2F1 are positively correlated in TCGA colon adenocarcinoma (COAD).

To determine the molecular mechanisms underlying the oncogenic function of USP2-AS1, we performed RNA-seq to profile gene expression in control and USP2-AS1 knockdown A549 cells. Knockdown of USP2-AS1 resulted in the downregulation of 929 genes and upregulation of 1115 genes (Supplementary Fig. S4A). These differentially expressed genes were subjected to Kyoto Encyclopedia of Genes and Genomes pathway enrichment analysis. Genes downregulated in USP2-AS1 knockdown cells were indeed enriched in cell cycle and cellular senescence pathways (Supplementary Fig. S4A). Intriguingly, gene set enrichment analysis revealed downregulation of E2F1 target genes in USP2-AS1 knockdown cells (Fig. 3A), indicating that USP2-AS1 positively regulates the E2F1 pathway. In support of this, ectopic expression of USP2-AS1 increased, whereas knockdown of USP2-AS1 decreased both mRNA and protein levels of E2F1 in A549 and HCT116 cells (Fig. 3B–I and Supplementary Fig. S4B–E). Moreover, analysis of the TCGA database showed that the expression levels of USP2-AS1 and E2F1 are positively correlated in COAD, READ, BRCA, and STAD (Fig. 3J and Supplementary Fig. S4F–I). These data collectively suggest that USP2-AS1 positively regulates E2F1 expression.

**Fig. 4:**
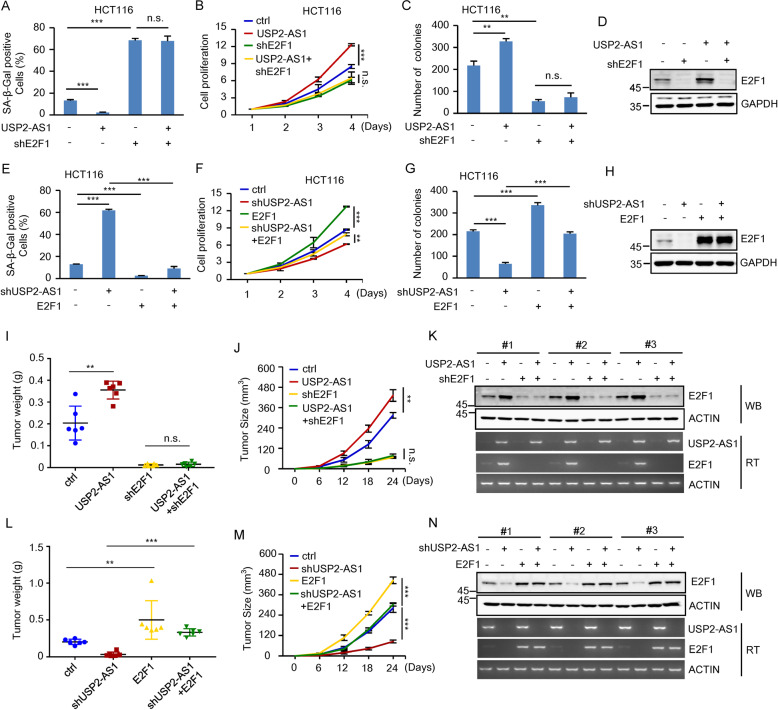
USP2-AS1 exerts its oncogenic role by increasing E2F1 expression. **A** HCT116 cells were infected with lentiviruses expressing control, USP2-AS1, E2F1 shRNA, or both USP2-AS1 and E2F1 shRNA as indicated. Ninety-six hours later, cells were subjected to β-galactosidase staining. Data shown are mean ± SD (*n* = 3). ***P < 0.001; n.s., no significance. The relative expression levels of USP2-AS1 were also shown in Supplementary Fig. S5A. **B** Shown are the growth curves of HCT116 cells expressing control, USP2-AS1, E2F1 shRNA, or both USP2-AS1 and E2F1 shRNA. Data shown are mean ± SD (*n* = 3). ***P < 0.001; n.s., no significance. **C** Colonies of HCT116 cells expressing control, USP2-AS1, E2F1 shRNA, or both USP2-AS1 and E2F1 shRNA were stained with crystal violet after 8 days of incubation. Data shown are mean ± SD (*n* = 3). **P < 0.01; n.s., no significance. **D** Lysates from HCT116 cells expressing control, USP2-AS1, E2F1 shRNA, or both USP2-AS1 and E2F1 shRNA were analyzed by western blotting. **E** HCT116 cells were infected with lentiviruses expressing control, USP2-AS1 shRNA, E2F1, or both USP2-AS1 shRNA and E2F1 as indicated. Ninety-six hours later, cells were subjected to β-galactosidase staining. Data shown are mean ± SD (*n* = 3). ***P < 0.001. The relative expression levels of USP2-AS1 were also shown in Supplementary Fig. S5G. **F** Shown are the growth curves of HCT116 cells expressing control, USP2-AS1 shRNA, E2F1, or both USP2-AS1 shRNA and E2F1. Data shown are mean ± SD (*n* = 3). **P < 0.01 and ***p < 0.001. **G** Colonies of HCT116 cells expressing control, USP2-AS1 shRNA, E2F1, or both USP2-AS1 shRNA and E2F1 were stained with crystal violet after 8 days of incubation. Data shown are mean ± SD (*n* = 3). ***P < 0.001. **H** Lysates from HCT116 cells expressing control, USP2-AS1 shRNA, E2F1, or both USP2-AS1 shRNA and E2F1 were analyzed by western blotting. **I**–**K** A total of 2 × 10^6^ HCT116 cells expressing control, USP2-AS1, E2F1 shRNA, or both USP2-AS1 and E2F1 shRNA were individually injected to the left and right flanks of nude mice (*n* = 6 for each group). Twenty-four days after injection, the xenografts were excised and weighed (**I**). Tumor sizes were measured at the indicated time points (**J**). RNA and protein extracts from the excised xenografts were also analyzed by RT-PCR and western blotting, respectively (**K**). **P < 0.01; n.s., no significance. RNA extracts were also analyzed by real-time RT-PCR (Supplementary Fig. S5N). **L**–**N** A total of 2 × 10^6^ HCT116 cells expressing either control, USP2-AS1 shRNA, E2F1, or both USP2-AS1 shRNA and E2F1 were individually injected to the left and right flanks of nude mice (*n* = 6 for each group). Twenty-four days after injection, the xenografts were excised and weighed (**L**). Tumor sizes were measured at the indicated time points (**M**). RNA and protein extracts from the excised xenografts were also analyzed by RT-PCR and western blotting, respectively (**N**). **P < 0.01 and ***p < 0.001. RNA extracts were also analyzed by real-time RT-PCR (Supplementary Fig. S5P).

We next performed the rescue experiments to examine whether USP2-AS1 functions via the positive regulation of E2F1. Ectopic expression of USP2-AS1 in HCT116 and A549 cells consistently decreased cellular senescence, accelerated cell proliferation, and increased the number of colonies (Fig. 4A–D and Supplementary Fig. S5A–F). However, USP2-AS1 failed to show any of these effects when E2F1 was knocked down (Fig. 4A–D and Supplementary Fig. S5A–F). In addition, the increased cellular senescence, the reduced cell proliferation, and the decreased number of colonies, caused by USP2-AS1 knockdown, could be markedly rescued by ectopically expressed E2F1 (Fig. 4E–H and Supplementary Fig. S5G–L). By using a xenograft mouse model, we showed that ectopic expression of USP2-AS1 promoted in vivo xenograft tumor growth of control cells, but not E2F1 knockdown cells (Fig. 4I–K and Supplementary Fig. S5M, N). Moreover, the inhibitory effect of USP2-AS1 knockdown on in vivo xenograft tumor growth was greatly reversed by E2F1 induction (Fig. 4L–N and Supplementary Fig. S5O, P). Taken together, these data suggest that USP2-AS1 exerts its oncogenic function by increasing E2F1 expression.

### USP2-AS1 promotes E2F1 mRNA stability via interaction with G3BP1

**Fig. 5:**
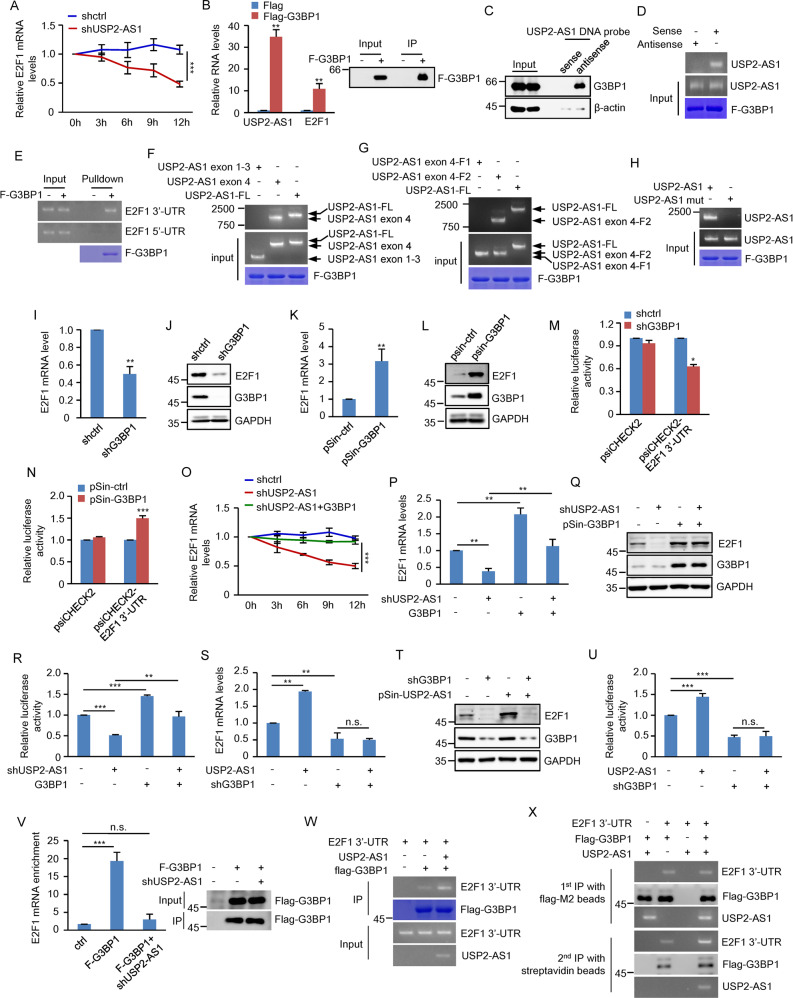
USP2-AS1 promotes E2F1 mRNA stability via interaction with G3BP1. **A** A549 cells expressing either control shRNA or USP2-AS1 shRNA were incubated with actinomycin D (2 μg/ml) for the indicated periods of time. Total RNA was then analyzed by real-time RT-PCR to examine E2F1 mRNA stability. Data shown are mean ± SD (*n* = 3). ***P < 0.001. **B** Lysates from A549 cells expressing control or Flag-G3BP1 proteins were immunoprecipitated with anti-Flag antibody. RNAs in immunoprecipitates were then analyzed by real-time RT-PCR to examine USP2-AS1 and E2F1 levels. Data shown are mean ± SD (*n* = 3). **P < 0.01. The input and immunoprecipitates were also analyzed by western blotting with anti-Flag antibody. **C** Lysates from A549 cells were incubated with either sense or antisense biotin-labeled DNA oligomers corresponding to USP2-AS1, followed by the pull-down experiments using streptavidin-coated beads. The pull-down complexes were analyzed by western blotting. **D** In vitro synthesized USP2-AS1 or its antisense RNA was incubated with purified recombinant Flag-G3BP1 bound with M2 beads. The beads-bound RNAs were then eluted as templates for RT-PCR analysis. **E** In vitro synthesized 5′-UTR or 3′-UTR of E2F1 was incubated with purified recombinant Flag-G3BP1 bound with M2 beads. After incubation and extensive washing, the beads-bound RNAs were eluted as templates for RT-PCR analysis. **F** In vitro synthesized USP2-AS1, USP2-AS1 exons 1–3, or USP2-AS1 exon 4 was incubated with purified recombinant Flag-G3BP1 bound with M2 beads. After incubation and extensive washing, the beads-bound RNAs were eluted as templates for RT-PCR analysis. **G** In vitro synthesized USP2-AS1, USP2-AS1 exon 4-F1, or USP2-AS1 exon 4-F2 was incubated with purified recombinant Flag-G3BP1 bound with M2 beads. After incubation and extensive washing, the beads-bound RNAs were eluted as templates for RT-PCR analysis. **H** In vitro synthesized USP2-AS1 or USP2-AS1 mutant (ΔG3BP1 BS) was incubated with purified recombinant Flag-G3BP1 bound with M2 beads. After incubation and extensive washing, the beads-bound RNAs were eluted as templates for RT-PCR analysis. **I**, **J** Total RNA (**I**) and lysates (**J**) from A549 cells expressing control shRNA or G3BP1 shRNA were analyzed by real-time RT-PCR and western blotting to examine E2F1 mRNA and protein levels, respectively. **P < 0.01. **K**, **L** Total RNA (**K**) and lysates (**L**) from A549 cells expressing control or Flag-G3BP1 were analyzed by real-time RT-PCR and western blotting to examine E2F1 mRNA and protein levels, respectively. **P < 0.01. **M** A549 cells expressing control shRNA or G3BP1 shRNA were transfected with psi-E2F1-3′-UTR or control construct. Twenty-four hours later, reporter activity was measured and plotted after normalizing with respect to Renilla luciferase activity. Data shown are mean ± SD (*n* = 3). *P < 0.05. **N** A549 cells expressing control or G3BP1 were transfected with psi-E2F1-3′-UTR or control construct. Twenty-four hours later, reporter activity was measured and plotted after normalizing with respect to Renilla luciferase activity. Data shown are mean ± SD (*n* = 3). ***P < 0.001. **O** A549 cells expressing either control, USP2-AS1 shRNA, or both USP2-AS1 shRNA and G3BP1 were incubated with actinomycin D (2 μg/ml) for the indicated periods of time. Total RNA was then analyzed by real-time RT-PCR to examine E2F1 mRNA stability. Data shown are mean ± SD (*n* = 3). ***P < 0.001. **P**, **Q** Total RNA (**P**) and lysates (**Q**) from A549 cells expressing control, USP2-AS1 shRNA, G3BP1, or both USP2-AS1 shRNA and G3BP1 were analyzed by real-time RT-PCR and western blotting to examine E2F1 mRNA and protein levels, respectively. **P < 0.01; n.s., no significance. The relative expression levels of USP2-AS1 were also shown in Supplementary Fig. S6G. **R** A549 cells expressing control, USP2-AS1 shRNA, G3BP1, or both USP2-AS1 shRNA and G3BP1 were transfected with psi-E2F1-3′-UTR construct. Twenty-four hours later, reporter activity was measured and plotted after normalizing with respect to Renilla luciferase activity. Data shown are mean ± SD (*n* = 3). **P < 0.01 and ***p < 0.001. **S**, **T** Total RNA (**S**) and lysates (**T**) from A549 cells expressing control, USP2-AS1, G3BP1 shRNA, or both USP2-AS1 and G3BP1 shRNA were analyzed by real-time RT-PCR and western blotting to examine E2F1 mRNA and protein levels, respectively. **P < 0.01; n.s., no significance. The relative expression levels of USP2-AS1 were also shown in Supplementary Fig. S6H. **U** A549 cells expressing control, USP2-AS1, G3BP1 shRNA, or both USP2-AS1 and G3BP1 shRNA were transfected with psi-E2F1-3′-UTR construct. Twenty-four hours later, reporter activity was measured and plotted after normalizing with respect to Renilla luciferase activity. Data shown are mean ± SD (*n* = 3). ***P < 0.001; n.s., no significance. **V** A549 cells expressing control shRNA or USP2-AS1 shRNA were transfected with or without Flag-G3BP1 as indicated. Twenty-four hours after transfection, cell lysates were immunoprecipitated with anti-Flag antibody. RNAs present in immunoprecipitates were then analyzed by real-time RT-PCR. Data shown are mean ± SD (*n* = 3). ***P < 0.001; n.s., no significance. The input and immunoprecipitates were also analyzed by western blotting with anti-Flag antibody. **W** Purified recombinant Flag-G3BP1 bound with M2 beads was incubated with in vitro synthesized E2F1 3′-UTR and USP2-AS1 in the indicated combinations. After incubation and extensive washing, the beads-bound RNAs were eluted as templates for RT-PCR analysis. **X** In vitro synthesized biotin-labeled E2F1 3′-UTR, unlabeled USP2-AS1 and Flag-G3BP1 were incubated at room temperature for 30 min in the indicated combinations. The mixtures were first immunoprecipitated with Flag-M2 beads, followed by the elution step with 3× FLAG peptides. Half of the eluent was subjected to western blot and RT-PCR analysis. The rest of the eluent was further incubated with streptavidin beads. After extensive washing, the pull-down precipitates were analyzed by western blotting and RT-PCR.

We next investigated how USP2-AS1 increases E2F1 expression. The primary localization of USP2-AS1 in the cytoplasm prompted us to ask whether USP2-AS1 could regulate E2F1 mRNA stability. Knockdown of USP2-AS1 reduced, whereas overexpression of USP2-AS1 prolonged, the half-life of E2F1 mRNA (Fig. 5A and Supplementary Fig. S6A), indicating that USP2-AS1 stabilizes E2F1 mRNA. To determine how E2F1 mRNA is stabilized by USP2-AS1, we sought to identify USP2-AS1-interacting proteins. Proteins pulled down by sense and antisense DNA oligomers corresponding to USP2-AS1 were separated by SDS-polyacrylamide gel electrophoresis (SDS-PAGE), followed by mass spectrometry analysis. A unique protein band with a molecular weight of approximately 66 kDa present in the USP2-AS1 antisense DNA oligomer precipitates was identified as G3BP1 (Supplementary Fig. S6B). This attracted our particular attention because as an RNA-binding protein, G3BP1 has been previously shown to have both mRNA-stabilizing and mRNA-degrading effects [43].

To validate the interaction of G3BP1 with USP2-AS1, we first performed a RIP experiment. USP2-AS1 was readily detected in the Flag-G3BP1 immunoprecipitates, but not in the control immunoprecipitates (Fig. 5B). The G3BP1-USP2-AS1 interaction was also verified by a biotin pull-down assay using a biotin-labeled antisense DNA oligomer against USP2-AS1 (Fig. 5C). Moreover, an in vitro binding assay showed that G3BP1 directly interacted with USP2-AS1, but not its antisense RNA (Fig. 5D). In addition to the interaction with USP2-AS1, G3BP1 was also shown to bind to the 3′-UTR of E2F1 mRNA (Fig. 5B, E). To further delineate the regions of USP2-AS1 that are responsible for its interaction with G3BP1, we generated a panel of USP2-AS1 deletion mutants and performed an in vitro binding assay (Supplementary Fig. S6C). Similar to full-length USP2-AS1, USP2-AS1 exon 4, and USP2-AS1 exon 4-F2 were strongly associated with G3BP1 (Fig. 5F, G). In contrast, USP2-AS1 exons 1–3 and USP2-AS1 exon 4-F1 showed no interaction with G3BP1 (Fig. 5F, G). These data indicate that the F2 region of USP2-AS1 exon 4 mediates the interaction with G3BP1. After careful inspection of this F2 region, two putative G3BP1-binding sites [44] were identified (Supplementary Fig. S6D). When these binding sites were deleted (ΔG3BP1 BS), the USP2-AS1-G3BP1 interaction was completely disrupted (Fig. 5H and Supplementary Fig. S6D). Together, these data demonstrate G3BP1 as a binding partner for USP2-AS1.

To determine whether USP2-AS1 promotes E2F1 mRNA stability via G3BP1, we first evaluated the effect of G3BP1 on E2F1 expression. Knockdown of G3BP1 decreased, whereas ectopic expression of G3BP1 increased, both mRNA and protein levels of E2F1 (Fig. 5I–L). In accordance with these results, luciferase expression from the reporter construct containing the 3′-UTR of E2F1 was reduced by G3BP1 knockdown, but induced by G3BP1 overexpression (Fig. 5M, N and Supplementary Fig. S6E). We next measured E2F1 mRNA half-life using actinomycin D. USP2-AS1 knockdown-decreased E2F1 mRNA stability could be restored by ectopically expressed G3BP1 (Fig. 5O). In addition, unlike the stabilizing effect of USP2-AS1 on E2F1 mRNA in control cells (Supplementary Fig. S6A), USP2-AS1 failed to increase E2F1 mRNA stability in G3BP1 knockdown cells (Supplementary Fig. S6F). In accordance, the reduced mRNA and protein expression of E2F1, as well as decreased luciferase expression from the E2F1 3′-UTR reporter construct, caused by USP2-AS1 knockdown could be markedly restored by ectopic expression of G3BP1 (Fig. 5P–R and Supplementary Fig. S6G). Moreover, USP2-AS1 overexpression was able to increase both mRNA and protein levels of E2F1 and induce luciferase expression from the E2F1 3′-UTR reporter construct in control cells, but not in G3BP1 knockdown cells (Fig. 5S–U and Supplementary Fig. S6H). Consistent with the above finding of no G3BP1-binding ability of USP2-AS1 mutant (ΔG3BP1 BS) (Fig. 5H), this mutant exhibited no obvious effect on E2F1 expression (Supplementary Fig. S6I, J). These data collectively indicate that the stabilizing effect of USP2-AS1 on E2F1 mRNA is dependent on G3BP1.

We next asked whether USP2-AS1 could facilitate the binding of G3BP1 to E2F1 3′-UTR and thereby stabilize E2F1 mRNA. A RIP experiment showed that knockdown of USP2-AS1 dramatically decreased the binding of G3BP1 to E2F1 mRNA (Fig. 5V), indicating the promoting effect of USP2-AS1 on the G3BP1-E2F1 mRNA interaction. In support of this, an in vitro binding assay revealed that USP2-AS1 was indeed able to enhance the interaction between G3BP1 and E2F1 3′-UTR (Fig. 5W). Moreover, the sequential immunoprecipitation experiment showed that USP2-AS1, G3BP1, and E2F1 3′-UTR could form a ternary complex (Fig. 5X). Taken together, these findings suggest that USP2-AS1 cooperates with G3BP1 to promote E2F1 mRNA stability.

### USP2-AS1 functions via G3BP1

**Fig. 6:**
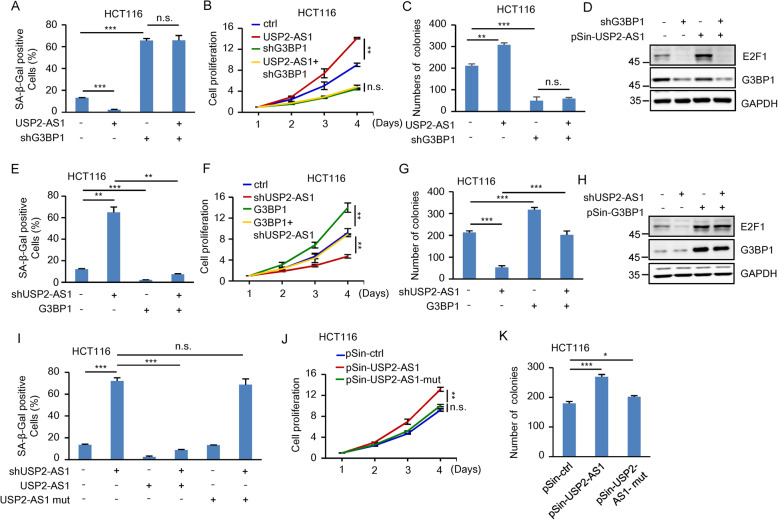
USP2-AS1 functions via G3BP1. **A** HCT116 cells were infected with lentiviruses expressing control, USP2-AS1, G3BP1 shRNA, or both USP2-AS1 and G3BP1 shRNA as indicated. Ninety-six hours later, cells were subjected to β-galactosidase staining. Data shown are mean ± SD (*n* = 3). ***P < 0.001; n.s., no significance. The relative expression levels of USP2-AS1 were also shown in Supplementary Fig. S7A. **B** Shown are the growth curves of HCT116 cells expressing control, USP2-AS1, G3BP1 shRNA, or both USP2-AS1 and G3BP1 shRNA. Data shown are mean ± SD (*n* = 3). **P < 0.01; n.s., no significance. **C** Colonies of HCT116 cells expressing control, USP2-AS1, G3BP1 shRNA, or both USP2-AS1 and G3BP1 shRNA were stained with crystal violet after 8 days of incubation. Data shown are mean ± SD (*n* = 3). **P < 0.01 and ***p < 0.001; n.s., no significance. **D** Lysates from HCT116 cells expressing control, USP2-AS1, G3BP1 shRNA, or both USP2-AS1 and G3BP1 shRNA were analyzed by western blotting. **E** HCT116 cells were infected with lentiviruses expressing control, USP2-AS1 shRNA, G3BP1, or both USP2-AS1 shRNA and G3BP1 as indicated. Ninety-six hours later, cells were subjected to β-galactosidase staining. Data shown are mean ± SD (*n* = 3). **P < 0.01 and ***p < 0.001. The relative expression levels of USP2-AS1 were also shown in Supplementary Fig. S7F. **F** Shown are the growth curves of HCT116 cells expressing control, USP2-AS1 shRNA, G3BP1, or both USP2-AS1 shRNA and G3BP1. Data shown are mean ± SD (*n* = 3). **P < 0.01. **G** Colonies of HCT116 cells expressing control, USP2-AS1 shRNA, G3BP1, or both USP2-AS1 shRNA and G3BP1 were stained with crystal violet after 8 days of incubation. Data shown are mean ± SD (*n* = 3). ***P < 0.001. **H** Lysates from HCT116 cells expressing control, USP2-AS1 shRNA, G3BP1, or both USP2-AS1 shRNA and G3BP1 were analyzed by western blotting. **I** HCT116 cells were infected with lentiviruses expressing control shRNA, USP2-AS1 shRNA, USP2-AS1 shRNA plus shRNA-resistant USP2-AS1, or USP2-AS1 shRNA plus shRNA-resistant USP2-AS1 mutant (ΔG3BP1 BS) as indicated. Ninety-six hours later, cells were subjected to β-galactosidase staining. Data shown are mean ± SD (*n* = 3). ***P < 0.001; n.s., no significance. The relative expression levels of USP2-AS1 were also shown in Supplementary Fig. S7K. **J** Shown are the growth curves of HCT116 cells expressing control, USP2-AS1, or USP2-AS1 mutant (ΔG3BP1 BS). Data shown are mean ± SD (*n* = 3). **P < 0.01; n.s., no significance. **K** Colonies of HCT116 cells expressing control, USP2-AS1, or USP2-AS1 mutant (ΔG3BP1 BS) were stained with crystal violet after 8 days of incubation. Data shown are mean ± SD (*n* = 3). *P < 0.05 and ***p < 0.001.

Given that the promoting effect of USP2-AS1 on E2F1 mRNA stability is G3BP1-dependent, we sought to evaluate whether USP2-AS1 functions via G3BP1. As was expected, knockdown of G3BP1 increased the senescence, reduced the proliferation, and decreased the colony number of both HCT116 and A549 cells (Fig. 6A–D and Supplementary Fig. S7A–E), while ectopic expression of G3BP1 in these cells showed the opposite effects (Fig. 6E–H and Supplementary Fig. S7F–J). USP2-AS1 overexpression consistently decreased cellular senescence, accelerated cell proliferation, and increased the number of colonies in control HCT116 and A549 cells (Fig. 6A–D and Supplementary Fig. S7A–E). However, USP2-AS1 failed to show any of these effects when G3BP1 was knocked down (Fig. 6A–D and Supplementary Fig. S7A–E). In addition, the increased cellular senescence, the reduced cell proliferation, and the decreased number of colonies caused by USP2-AS1 knockdown could be markedly rescued by ectopically expressed G3BP1 (Fig. 6E–H and Supplementary Fig. S7F–J). These data indicate the importance of G3BP1 in mediating USP2-AS1 function.

To further support this idea, the G3BP1-binding-defective mutant of USP2-AS1 (ΔG3BP1 BS) was used. Correlating with the above findings that USP2-AS1 (ΔG3BP1 BS) was not able to increase E2F1 expression (Supplementary Fig. S6I, J), this mutant USP2-AS1 failed to reverse the USP2-AS1 knockdown-induced senescence of HCT116 and A549 cells (Fig. 6I and Supplementary Fig. S7K–M). Moreover, unlike wild-type USP2-AS1, this mutant USP2-AS1 was also not capable of increasing cell proliferation and the number of colonies (Fig. 6J, K and Supplementary Fig. S7N, O). Together, these findings suggest that USP2-AS1 exerts its cellular function via G3BP1.

### USP2-AS1 is a mediator of the oncogenic function of c-Myc

**Fig. 7:**
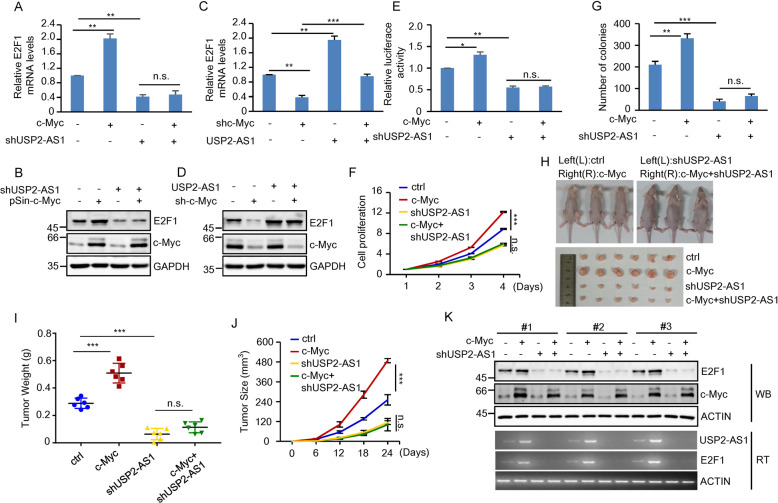
USP2-AS1 is a mediator of the oncogenic function of c-Myc. **A**, **B** Total RNA (**A**) and lysates (**B**) from HCT116 cells expressing control, c-Myc, USP2-AS1 shRNA, or both c-Myc and USP2-AS1 shRNA were analyzed by real-time RT-PCR and western blotting, respectively. **P < 0.01; n.s., no significance. The relative expression levels of USP2-AS1 were also shown in Supplementary Fig. S8A. **C**, **D** Total RNA (**C**) and lysates (**D**) from HCT116 cells expressing control, c-Myc shRNA, USP2-AS1, or both c-Myc shRNA and USP2-AS1 were analyzed by real-time RT-PCR and western blotting, respectively. **P < 0.01 and ***p < 0.001. The relative expression levels of USP2-AS1 were also shown in Supplementary Fig. S8B. **E** HCT116 cells expressing control, c-Myc, USP2-AS1 shRNA, or both c-Myc and USP2-AS1 shRNA were transfected with psi-E2F1-3′-UTR. Twenty-four hours later, reporter activity was measured and plotted after normalizing with respect to Renilla luciferase activity. Data shown are mean ± SD (*n* = 3). *P < 0.05; **p < 0.01; n.s., no significance. **F** Shown are the growth curves of HCT116 cells expressing control, c-Myc, USP2-AS1 shRNA, or both c-Myc and USP2-AS1 shRNA. Data shown are mean ± SD (*n* = 3). ***P < 0.001; n.s., no significance. **G** Colonies of HCT116 cells expressing control, c-Myc, USP2-AS1 shRNA, or both c-Myc and USP2-AS1 shRNA were stained with crystal violet after 8 days of incubation. Data shown are mean ± SD (*n* = 3). **P < 0.01; ***p < 0.001; n.s., no significance. **H**–**K** A total of 2 × 10^6^ HCT116 cells expressing control, c-Myc, USP2-AS1 shRNA, or both c-Myc and USP2-AS1 shRNA were individually injected to the left and right flanks of nude mice as indicated (*n* = 6 for each group). Representative photographs of mice and xenograft tumors were taken 24 days after injection (**H**). The excised tumors were weighed (**I**). Tumor sizes were measured at the indicated time points (**J**). RNA and protein extracts from the excised xenografts were also analyzed by RT-PCR and western blotting, respectively (**K**). ***P < 0.001; n.s., no significance. RNA extracts were also analyzed by real-time RT-PCR (Supplementary Fig. S8D).

It has been previously reported that c-Myc is able to positively regulate E2F1 expression [20, 45]. Given the above findings that USP2-AS1 is transcriptionally upregulated by c-Myc and USP2-AS1 is capable of increasing E2F1 expression, we sought to examine whether USP2-AS1 is involved in c-Myc-induced E2F1 expression. As was expected, c-Myc overexpression increased both mRNA and protein levels of E2F1 in HCT116 cells (Fig. 7A, B and Supplementary Fig. S8A). However, this promoting effect of c-Myc on E2F1 expression was greatly compromised by USP2-AS1 knockdown (Fig. 7A, B and Supplementary Fig. S8A). In addition, c-Myc knockdown-decreased mRNA and protein levels of E2F1 could be markedly restored by USP2-AS1 overexpression (Fig. 7C, D and Supplementary Fig. S8B). Moreover, c-Myc-induced luciferase expression from the E2F1 3′-UTR reporter construct was strongly reversed by the knockdown of USP2-AS1 (Fig. 7E). These data indicate the involvement of USP2-AS1 in c-Myc-induced E2F1 expression.

We next investigated whether USP2-AS1 could mediate the oncogenic function of c-Myc. Ectopic expression of c-Myc was expectedly shown to increase the proliferation, colony number, and Edu-positive cell number of HCT116 cells (Fig. 7F, G and Supplementary Fig. S8C). However, c-Myc showed no effect on the proliferation, colony number, and Edu-positive cell number when USP2-AS1 was knocked down in these cells (Fig. 7F, G and Supplementary Fig. S8C). By using a xenogaft mouse model, we found that ectopically expressed c-Myc in HCT116 cells greatly accelerated in vivo xenograft tumor growth (Fig. 7H–J). Accompanying the accelerated tumor growth by c-Myc overexpression, expression levels of both USP2-AS1 and E2F1 were elevated in xenografts generated from c-Myc-overexpresed HCT116 cells (Fig. 7K and Supplementary Fig. S8D), indicating that c-Myc may promote tumor growth via USP2-AS1. In support of this, USP2-AS1 knockdown significantly minimized the promoting effect of c-Myc on in vivo xenograft tumor growth (Fig. 7H–J). We should mention that in this tumor xenograft experiment, we used six mice for each group. Given that different experimental conditions were not conducted on the same mouse, it would be better to use a larger amount of mice in each condition. Taken together, these data suggest that USP2-AS1 is an important mediator of the oncogenic function of c-Myc via the regulation of E2F1.

## Discussion

The *c-Myc* gene is one of the most frequently deregulated oncogenes and a driver for a variety of human cancers [16, 19]. As a transcription factor, c-Myc regulates multiple cellular processes including cell proliferation, differentiation, metabolism, and senescence by modulating target gene expression. LncRNAs are emerging as either effectors or regulators of the c-Myc signaling [46–51]. LncRNAs are also involved in the regulation of cellular senescence [46, 52–54]. However, it is unclear whether c-Myc could exert its tumor-promoting effect via senescence-regulatory lncRNAs. In this study, we report that USP2-AS1, a c-Myc-inducible lncRNA, is able to inhibit cellular senescence and mediate the oncogenic role of c-Myc by promoting E2F1 mRNA stability. Therefore, USP2-AS1 appears to be an important regulator of c-Myc function.

The *USP2-AS1* gene is located head to head with the *USP2* gene on chromosome 11. By analyzing the RNA-seq data and performing validation experiments, USP2-AS1 is shown to be primarily expressed as a 2486 nt RNA transcript and predominantly localized in the cytoplasm. USP2-AS1 has been recently reported to facilitate the progression of ovarian and colon tumors [55, 56]. Consistent with these reports, we here show that USP2-AS1 functions as an oncogenic molecule. We also provide evidences demonstrating that USP2-AS1 is a bona fide transcriptional target of c-Myc. The finding of the positive correlation of c-Myc and USP2-AS1 levels in COAD, READ, BRCA, PRAD, and STAD further indicates the importance of c-Myc-regulated USP2-AS1 expression.

Cellular senescence is a state of stable cell cycle arrest. This cell cycle exit is intricately controlled by the p53/p21 and p16/Rb tumor-suppressive pathways [10]. As the potent inhibitors of CDKs, p21 and p16 are able to inhibit CDK-dependent Rb phosphorylation, thereby resulting in E2F1 inhibition and the subsequent cell cycle arrest [57]. Correlating with the effect of E2F1 on cell cycle progression, E2F1 can also regulate cellular senescence [58]. While overexpression of E2F1 induces senescence in normal cells [59], suppression of E2F1 triggers senescence in cancer and immortalized cells [60–62], indicating the complexity of the regulation of cellular senescence by E2F1. In this study, we clearly show that by increasing E2F1 expression, USP2-AS1 negatively regulates senescence in both HCT116 and A549 cells. The physiological importance of USP2-AS1-increased E2F1 expression is also supported by the observation that USP2-AS1 and E2F1 expression levels are positively correlated in COAD, READ, BRCA, PRAD, and STAD. Interestingly, USP2-AS1 appears to be able to negatively regulate p53 expression (Supplementary Fig. 8E, F), yet the underlying mechanism needs to be determined in the future. Therefore, it is possible that USP2-AS1 could inhibit senescence through the regulation of both E2F1 and p53.

It has been previously shown that c-Myc activates the transcription of E2F1, while c-Myc-activated miR-17-5p and miR-20a suppress E2F1 translation [20, 45, 63], indicating the tight regulation of the c-Myc-E2F1 network in cells. We here show that USP2-AS1 is able to increase E2F1 mRNA stability and participate in c-Myc-induced E2F1 expression. Together with the recent finding of the involvement of the lncRNA EMS in c-Myc-increased E2F1 expression [40], our data suggest that lncRNA is an important class of regulatory molecule that finely controls the c-Myc-E2F1 network.

Increasing evidence suggests that lncRNA regulates gene expression via diverse mechanisms [64]. Of note, lncRNA could post-transcriptionally modulate gene expression through interaction with protein [65]. In this study, we reveal that USP2-AS1 cooperates with the RNA-binding protein G3BP1 to promote E2F1 mRNA stability and in turn increase E2F1 expression. G3BP1 is best known for its role in the regulation of the assembly and dynamics of stress granules [66–68]. G3BP1 also exhibits both mRNA-stabilizing and mRNA-degrading effects [43, 69]. Overexpression of G3BP1 has been implicated in a variety of human cancers [70], indicating the oncogenic function of G3BP1. In support, G3BP1 is herein shown to interact with E2F1 3′-UTR and increase E2F1 mRNA stability, thereby leading to suppressed cellular senescence and accelerated cell proliferation. We also identify USP2-AS1 as a binding partner for G3BP1. The putative G3BP1-binding sites within exon 4 of USP2-AS1 appear to be required for the interaction with G3BP1. By binding to G3BP1, USP2-AS1 is able to facilitate the interaction of G3BP1 to E2F1 3′-UTR. Moreover, USP2-AS1, G3BP1, and E2F1 3′-UTR could form a ternary complex. These findings suggest that USP2-AS1 may act as a scaffold to promote the interaction between G3BP1 and E2F1 3′-UTR, although the detailed underlying mechanisms need to be further examined. Functionally, USP2-AS1 indeed increases E2F1 mRNA stability and exerts its function via G3BP1. Together with the previous finding that the lncRNA P53RRA interacts with G3BP1 and promotes ferroptosis and apoptosis [71], our data, therefore, indicate G3BP1 as an important regulator of lncRNA’s cellular function.

It has been well recognized that activation of c-Myc is not only able to drive tumor initiation, progression, and recurrence but is also necessary for tumor maintenance [16], making c-Myc an attractive target for anticancer therapy. Although c-Myc is traditionally considered as a difficult-to-drug target, several compounds that directly or indirectly target c-Myc have recently been reported to exhibit anticancer activity in preclinical tumor models [72, 73]. In the present study, we show that as a direct transcriptional target of c-Myc, USP2-AS1 suppresses cellular senescence and functions as an oncogenic lncRNA, indicating that USP2-AS1 may represent a potential target for anticancer therapy. However, although we here provide evidences showing that USP2-AS1 acts as a mediator of the oncogenic function of c-Myc, we believe that c-Myc functions as an oncoprotein via regulating different target gene expression, because c-Myc is a master transcription factor.

## Supplementary information

Table S1

Table S2

Table S3

Supplementary Figures and Legends

## Data Availability

The RNA-sequencing data have been deposited in the National Center for Biotechnology Information (NCBI) Gene Expression Omnibus (GEO) with accession code GSE169138.
