# Supplementary material for: c-Myc-activated USP2-AS1 suppresses senescence and promotes tumor progression via stabilization of E2F1 mRNA
Source: Cell Death Dis. 2021 Oct 27;12(11):1006. doi: 10.1038/s41419-021-04330-2 (PMC8551278; doi:10.1038/s41419-021-04330-2)
Supplement: Supplementary file 1 — Table S1 [file 41419_2021_4330_MOESM1_ESM.docx]

| **Primers used in qRT-PCR assays** | |
| --- | --- |
| β-Actin | Fw:5'GACCTGACTGACTACCTCATGAAGAT3' |
|  | Rev:5'GTCACACTTCATGATGGAGTTGAAGG3' |
| GAPDH | Fw: 5'CCATGGGGAAGGTGAAGGTC3' |
|  | Rev: 5'GAAGGGGTCATTGATGGCAAC3' |
| U1 | Fw: 5'GGGAGATACCATGATCACGAAGGT3' |
|  | Rew: 5'CCACAAATTATGCAGTCGAGTTTCCC3' |
| E2F1(CDS) | Fw: 5'GCCACTGACTCTGCCACCATAG3' |
|  | Rev: 5'CTGCCCATCCGGGACAAC3' |
| USP2-AS1 | Fw: 5'TTCAGCCCTGTCATCTTGTG3' |
|  | Rev: 5'GGTCTTGAGCACCTCAATTCT3' |
| G3BP1 | Fw: 5'GATGCAGTCTACGGACAGAAAG3' |
|  | Rev: 5'TGGACTACCACACCATCATTTAG3' |
| lncRNA-2 | Fw: 5' TGCTTCTCGAAACACCGCA3' |
|  | Rev: 5' TGCGCAAACCTTGATGAACC3' |
| lncRNA-3 | Fw: 5'ATCCCAGTACTTTGGGAGGT3' |
|  | Rev: 5'CACCACACCCAGCTCATTT3' |
| lncRNA-4 | Fw: 5'TGAGGACATGGGAAGGAAATG3' |
|  | Rev: 5'TTCTGGAGGCTGGAAGTCTA3' |
| lncRNA-5 | Fw: 5'GGAAACCCAAACCAAGGAGA3' |
|  | Rev: 5'CCAGGAGGTGCCCTATTAATTT3' |
| lncRNA-6 | Fw: 5'AGAGTGCAGTGTCCTGAATG3' |
|  | Rev: 5'GCCAGTGTAATTTCTGTCAACC3' |
| **Primers used for antisense oligomer affinity pull-down assays** | |
| USP2-AS1 sense oligo DNA | 5' (biotin-)GCTGGAATCCAGGAAGAATTG3' |
| USP2-AS1 antisense oligo DNA | 5' (biotin-)CAATTCTTCCTGGATTCCAGC3' |
| **Primers used in ChIP assays** | |
| BS1 | Fw: 5'GTCACGGCTATTGGCTTGTT3' |
|  | Rev: 5'CGTGTGACATTCCAGTCCAC3' |
| GAPDH promoter | Fw: 5'TACTAGCGGTTTTACGGGCG3' |
|  | Rev: 5'TCGAACAGGAGGAGCAGAGAGCGA3' |
| **RACE primers** | |
| 5'RACE-P1 | AGTCCAGTCTGGGCTGTGCAGGGCTGC |
| P2(UPM) | CTAATACGACTCACTATAGGGC |
| 3'RACE-P3 | CCAACCTGGGTGAGAGAGTGAGACCCAG |
| P4(UPM) | CTAATACGACTCACTATAGGGC |
| **Primers used in Semi-quantitative assays** | |
| USP2-AS1 | Fw: 5'ATACACAGAGAACCACGTGGA3' |
|  | Rev: 5'TACAGTTGCACGTGGCTAGGGAA3' |
| USP2-AS1 exon1-3 | Fw: 5'ATACACAGAGAACCACGTGGA3' |
|  | Rev: 5'TTTGCACAAGATGACAGGGCTGA3' |
| USP2-AS1 exon4 | Fw: 5'TCCTCACTGTCCCCAGAAGGAAG3' |
|  | Rev: 5'TACAGTTGCACGTGGCTAGGGAA3' |
| USP2-AS1 exon4-F1 | Fw: 5'TCCTCACTGTCCCCAGAAGGAAG3' |
|  | Rev: 5'TCCTCGAATTTAAGTATTCATTTTG3' |
| USP2-AS1 exon4-F2 | Fw: 5'AAAAATACATTCTTACACCTTTAAT3' |
|  | Rev: 5'TACAGTTGCACGTGGCTAGGGAA3' |
| E2F1-5’UTR | Fw: 5'GGGACTTTGCAGGCAG3' |
|  | Rev: 5'GACGCTCACGGCCCGCGCGG3' |
| E2F1-3’UTR | Fw: 5'CAGGGCTTGGAGGGACCA3' |
|  | Rev: 5'TTGGCTTTCTACAACAAA3' |
| **Oligonucleotide sequence of shRNAs** | |
| shUSP2-AS1#1 | 5'TAATCTCAGCGAGATGAAGAG3' |
| shUSP2-AS1#2 | 5'GCACTCAGTTGTACTTCTTTA3' |
| sh-c-Myc | 5'CTGAGACAGATCAGCAACAA3' |
| sh-G3BP1 | 5'GCCTGTAAGAAATACAGGATTCT3' |
| sh-E2F1 | 5'AGGATGGATATGAGATGGGA3' |
| sh-control | 5'CCTAAGGTTAAGTCGCCCTCG3' |
| shlncRNA-2 | 5'GCTTCTTGCTAGAGGATTTC3' |
| shlncRNA-3 | 5'GCCGGAGAACAAATGTTAA3' |
| shlncRNA-4 | 5'GGCACTTGAGCTCAAACAT3' |
| shlncRNA-5 | 5'GGGAATTCGGGCTGTGTTTA3' |
| **Primers for in vitro transcription** | |
| The T7 RNA polymerase sequence(T7) was  5'CCAAGCTTCTAATACGACTCACTATAGGGAGA 3' | |
| E2F1-5’UTR(sense) | 5' (T7)GGGACTTTGCAGGCAG3' |
|  | 5'GACGCTCACGGCCCGCGCGG3' |
| E2F1-3’UTR(sense) | 5' (T7)CAGGGCTTGGAGGGA3' |
|  | 5'ACCTTTACTGGATCTG3' |
| USP2-AS1(sense) | 5' (T7) ATACACAGAGAACCACGTGGAGGAAA3' |
|  | 5' TACAGTTGCACGTGGCTAGGGAA3' |
| USP2-AS1(antisense) | 5'ATACACAGAGAACCACGTGGAGGAAA3' |
|  | 5' (T7) TACAGTTGCACGTGGCTAGGGAA3' |
| USP2-AS1 exon1-3(sense) | 5' (T7) ATACACAGAGAACCACGTGGAGGAAA3' |
|  | 5' TTTGCACAAGATGACAGGGCTGA3' |
| USP2-AS1 exon1-3(antisense) | 5'ATACACAGAGAACCACGTGGA3' |
|  | 5' (T7) TTTGCACAAGATGACAGGGCTGA3' |
| USP2-AS1 exon4(sense) | 5' (T7) TCCTCACTGTCCCCAGAAG3' |
|  | 5' TACAGTTGCACGTGGCTAGGGAA 3' |
| USP2-AS1 exon4(antisense) | 5'TCCTCACTGTCCCCAGAAG3' |
|  | 5' (T7) TACAGTTGCACGTGGCTAGGGAA 3' |
| USP2-AS1 exon4-F1(sense) | 5' (T7) TCCTCACTGTCCCCAGAAG3' |
|  | 5'TCCTCGAATTTAAGTATTCATTTTGG 3' |
| USP2-AS1 exon4-F1(antisense) | 5'TCCTCACTGTCCCCAGAAG3' |
|  | 5' (T7) TCCTCGAATTTAAGTATTCATTTTGG3' |
| USP2-AS1 exon4-F2(sense) | 5' (T7) AAAAATACATTCTTACACCTTTAATT3' |
|  | 5' TTTGCACAAGATGACAGGGCTGA 3' |
| USP2-AS1 exon4-F2(antisense) | 5'AAAAATACATTCTTACACCTTTAATT3' |
|  | 5' (T7) TTTGCACAAGATGACAGGGCTGA3' |
