# Supplementary material for: c-Myc-activated USP2-AS1 suppresses senescence and promotes tumor progression via stabilization of E2F1 mRNA
Source: Cell Death Dis. 2021 Oct 27;12(11):1006. doi: 10.1038/s41419-021-04330-2 (PMC8551278; doi:10.1038/s41419-021-04330-2)
Supplement: Supplementary file 4 — Supplementary Figures and Legends [file 41419_2021_4330_MOESM4_ESM.pdf]

Figure S1. Related to Figure 1

(A) By analyzing our previously published c-Myc-induced lncRNA dataset (SRP171977) and ENCODE c-Myc chromatin immunoprecipitation sequencing (ChIP-seq) datasets, six potential c-Myc-responsive lncRNAs were chosen for further validation.

(B) Total RNA from A549 cells expressing control or c-Myc was analyzed by real-time RT-PCR to examine expression levels of the indicated lncRNAs. Data shown are mean ± SD (n=3). *, $p < 0.05$; **, $p < 0.01$.

(C and D) A549 cells were infected with lentiviruses expressing the indicated shRNAs. Ninety-six hours later, cells were subjected to β-galactosidase staining (C). The knockdown efficiency of the indicated RNAs was determined by real-time RT-PCR analysis (D).

(E and F) Our previously published RNA-seq data (SRP171977) were analyzed by Cutadapt v1.18 to remove adapters and low-quality reads. Clean reads were then aligned to human reference genome assembly version GRCh38/hg38 using STAR_2.6.1a and assembled by StringTie. All of the assemblies and the reference transcriptome annotation were merged by StringTie--merge. All of the aligned bam files were visualized in IGV (E). StringTie-e was used to calculate transcripts per million (TPM) of the indicated transcripts (F).

(G) Shown are products obtained from 5'-RACE (rapid amplification of cDNA ends) and 3'-RACE experiments. P1 and P2 were used for 5'-RACE. P3 and P4 were used for 3'-RACE.

(H) Total RNA from A549 and HCT116 cells was analyzed by RT-PCR using primers corresponding to the 5’ and 3’ ends of USP2-AS1.

(I and J) Both cytoplasmic and nuclear fractions from HCT116 (I) and A549 (J) cells were analyzed by real-time RT-PCR to determine USP2-AS1 localization.

Figure S2. Related to Figure 1

(A and B) HCT116 (A) and A549 (B) cells were infected with lentiviruses expressing control shRNA, USP2-AS1 shRNA#1, or USP2-AS1 shRNA#2. Ninety-six hours later,

cells were immunostained with anti-H3K9me3 antibody to examine senescence-associated heterochromatin foci (SAHF) formation. A minimum of 100 cells were counted in each condition. The number of SAHF per cell were calculated. Data shown are mean ± SD from three independent experiments. **, $p < 0.\ 01$; ***, $p < 0.001$.

(C) The knockdown efficiency of USP2-AS1 in A549 cells was verified by real-time RT-PCR analysis for Fig. 1A. ***, $p < 0.001$.

(D) The knockdown efficiency of USP2-AS1 in HCT116 cells was verified by real-time RT-PCR analysis for Fig. 1B. ***, $p < 0.001$.

(E and F) HCT116 cells were infected with lentiviruses expressing control shRNA, USP2-AS1 shRNA, USP2-AS1, or USP2-AS1 shRNA plus shRNA-resistant USP2-AS1. (E) Ninety-six hours later, cells were subjected to β-galactosidase staining. (F) Shown are the relative expression levels of USP2-AS1 in each condition. Data shown are mean ± SD (n=3). *, $p < 0.05$; ***, $p < 0.001$.

(G and H) A549 cells were infected with lentiviruses expressing control shRNA, USP2-AS1 shRNA, USP2-AS1, or USP2-AS1 shRNA plus shRNA-resistant USP2-AS1. (G) Ninety-six hours later, cells were subjected to β-galactosidase staining. (H) Shown are the relative expression levels of USP2-AS1 in each condition. Data shown are mean ± SD (n=3). **, $p < 0.\ 01$; ***, $p < 0.001$; n.s., no significance.

(I) Shown are the growth curves of A549 cells expressing control shRNA, USP2-AS1 shRNA#1, or USP2-AS1 shRNA#2. Data shown are mean ± SD (n=3). ***, $p < 0.001$.

(J) Colonies of A549 cells expressing control shRNA, USP2-AS1 shRNA#1, or USP2-AS1 shRNA#2 were stained with crystal violet after 12 days of incubation. The shown images are representative of three independent experiments. Data shown are mean ± SD (n=3). ***, $p < 0.001$.

(K) The successful overexpression of USP2-AS1 in HCT116 cells was determined by real-time RT-PCR analysis for Fig.e 1J. ***, $p < 0.001$.

(L) Shown are the growth curves of A549 cells expressing control or USP2-AS1. Data shown are mean ± SD (n=3). **, $p < 0.01$.

(M) Colonies of A549 cells expressing control or USP2-AS1 were stained with crystal violet after 12 days of incubation. The shown images are representative of three

independent experiments. Data shown are mean ± SD (n=3). ***, $p < 0.001$.

(N) The successful overexpression of USP2-AS1 in A549 cells was determined by real-time RT-PCR analysis. ***, $p < 0.001$.

(O) RNA from the excised xenografts (Fig. 1L) was analyzed by real-time RT-PCR to examine relative USP2-AS1 levels.

(P) RNA from the excised xenografts (Fig. 1P) was analyzed by real-time RT-PCR to examine relative USP2-AS1 levels.

Figure S3. Related to Figure 2

(A-D) The expression levels of c-Myc and USP2-AS1 are positively correlated in TCGA rectum adenocarcinoma (READ) (A), breast invasive carcinoma (BRCA) (B), prostate adenocarcinoma (PRAD) (C), and stomach adenocarcinoma (STAD) (D).

Figure S4. Related to Figure 3

(A) Volcano plot showing expression of 929 genes downregulated and 1115 genes upregulated after USP2-AS1 knockdown in A549 cells (2 biological replicates per group). Threshold of differential expression is adjusted p value of < 0.05 and fold change of > 2. These differentially expressed genes were also subjected to Kyoto Encyclopedia of Genes and Genomes (KEGG) pathway enrichment analysis.

(B) The successful overexpression of USP2-AS1 in A549 cells was verified by real-time RT-PCR analysis for Fig. 3B. ***, $p < 0.001$.

(C) The successful overexpression of USP2-AS1 in HCT116 cells was verified by real-time RT-PCR analysis for Fig. 3D. ***, $p < 0.001$.

(D) The successful knockdown of USP2-AS1 in A549 cells was verified by real-time RT-PCR analysis for Fig. 3F. ***, $p < 0.001$.

(E) The successful knockdown of USP2-AS1 in HCT116 cells was verified by real-time RT-PCR analysis for Fig. 3H. ***, $p < 0.001$.

(F-I) The expression levels of USP2-AS1 and E2F1 are positively correlated in TCGA rectum adenocarcinoma (READ) (F), breast invasive carcinoma (BRCA) (G), prostate adenocarcinoma (PRAD) (H), and stomach adenocarcinoma (STAD) (I).

Figure S5. Related to Figure 4

(A) The relative expression levels of USP2-AS1 were examined by real-time RT-PCR for Fig. 4A. ***, $p < 0.001$; n.s., no significance.

(B and C) A549 cells were infected with lentiviruses expressing control, USP2-AS1, E2F1 shRNA, or both USP2-AS1 and E2F1 shRNA as indicated. (B) Ninety-six hours later, cells were subjected to β-galactosidase staining. (C) Shown are the relative expression levels of USP2-AS1 in each condition. Data shown are mean ± SD (n=3). **, $p < 0.01$; ***, $p < 0.001$; n.s., no significance.

(D) Shown are the growth curves of A549 cells expressing control, USP2-AS1, E2F1 shRNA, or both USP2-AS1 and E2F1 shRNA. Data shown are mean ± SD (n=3). **, $p < 0.01$; n.s., no significance.

(E) Colonies of A549 cells expressing control, USP2-AS1, E2F1 shRNA, or both USP2-AS1 and E2F1 shRNA were stained with crystal violet after 12 days of incubation. Data shown are mean ± SD (n=3). ***, $p < 0.001$; n.s., no significance.

(F) Lysates from A549 cells expressing control, USP2-AS1, E2F1 shRNA, or both USP2-AS1 and E2F1 shRNA were analyzed by western blotting.

(G) The relative expression levels of USP2-AS1 were examined by real-time RT-PCR for Fig. 4E. ***, $p < 0.001$; n.s., no significance.

(H and I) A549 cells were infected with lentiviruses expressing control, USP2-AS1 shRNA, E2F1, or both USP2-AS1 shRNA and E2F1 as indicated. (H) Ninety-six hours later, cells were subjected to β-galactosidase staining. (I) Shown are the relative expression levels of USP2-AS1 in each condition. Data shown are mean ± SD (n=3). ***, $p < 0.001$.

(J) Shown are the growth curves of A549 cells expressing control, USP2-AS1 shRNA, E2F1, or both USP2-AS1 shRNA and E2F1. Data shown are mean ± SD (n=3). **, $p < 0.01$.

(K) Colonies of A549 cells expressing control, USP2-AS1 shRNA, E2F1, or both USP2-AS1 shRNA and E2F1 were stained with crystal violet after 12 days of incubation. Data shown are mean ± SD (n=3). ***, $p < 0.001$.

(L) Lysates from A549 cells expressing control, USP2-AS1 shRNA, E2F1, or both

USP2-AS1 shRNA and E2F1 were analyzed by western blotting.

(M) 2×10^6 HCT116 cells expressing either control, USP2-AS1, E2F1 shRNA, or both USP2-AS1 and E2F1 shRNA were individually injected to the left and right flanks of nude mice as indicated (n=6 for each group). Representative photographs of mice and xenograft tumors were taken twenty-four days after injection.

(N) RNA from the excised xenografts (M) was analyzed by real-time RT-PCR to examine relative USP2-AS1 levels.

(O) 2×10^6 HCT116 cells expressing either control, USP2-AS1 shRNA, E2F1, or both USP2-AS1 shRNA and E2F1 were individually injected to the left and right flanks of nude mice as indicated (n=6 for each group). Representative photographs of mice and xenograft tumors were taken twenty-four days after injection.

(P) RNA from the excised xenografts (O) was analyzed by real-time RT-PCR to examine relative USP2-AS1 levels.

Figure S6. Related to Figure 5

(A) A549 cells expressing either control or USP2-AS1 were incubated with actinomycin D (2μg/ml) for the indicated periods of time. Total RNA was then analyzed by real-time RT-PCR to examine E2F1 mRNA stability. Data shown are mean ± SD (n=3). **, $p < 0.01$.

(B) Lysates from A549 cells were incubated with either sense or antisense biotin-labeled DNA oligomers corresponding to USP2-AS1, followed by the pull-down experiments using streptavidin-coated beads. The pull-downed proteins were separated by SDS-PAGE and visualized by Coomassie brilliant blue staining. The separated proteins were analyzed by mass spectrometry. G3BP1 was identified as a potential USP2-AS1-binding protein. The G3BP1 peptide sequences obtained by MS are also shown.

(C) Illustration of full-length USP2-AS1 and its deletion mutants used in the mapping experiments.

(D) Illustration of USP-AS1 with two G3BP1 potential binding sites highlighted in green color. USP2-AS1 mut (ΔG3BP1 BS) indicates that these sites are deleted.

(E) Shown is the psiCHECK2-based E2F1 3'-UTR reporter construct used for luciferase assay.

(F) A549 cells expressing control, G3BP1 shRNA, or both USP2-AS1 and G3BP1 shRNA were incubated with actinomycin D (2μg/ml) for the indicated periods of time. Total RNA was then analyzed by real-time RT-PCR to examine E2F1 mRNA stability. Data shown are mean ± SD (n=3). ***, $p < 0.001$.

(G) The relative expression levels of USP2-AS1 were examined by real-time RT-PCR for Fig. 5P. ***, $p < 0.001$; n.s., no significance.

(H) The relative expression levels of USP2-AS1 were examined by real-time RT-PCR for Fig. 5S. ***, $p < 0.001$; n.s., no significance.

(I and J) Total RNA (I) and lysates (J) from A549 cells expressing control, USP2-AS1, or USP2-AS1 mutant (ΔG3BP1 BS) (D) were analyzed by real-time RT-PCR and western blotting to examine mRNA and protein levels of E2F1, respectively. **, $p < 0.01$; n.s., no significance.

Figure S7. Related to Figure 6

(A) The relative expression levels of USP2-AS1 were examined by real-time RT-PCR for Fig. 6A. ***, $p < 0.001$; n.s., no significance.

(B) A549 cells were infected with lentiviruses expressing control, USP2-AS1, G3BP1 shRNA, or both USP2-AS1 and G3BP1 shRNA as indicated. Ninety-six hours later, cells were subjected to β-galactosidase staining. Data shown are mean ± SD (n=3). **, $p < 0.01$; n.s., no significance.

(C) Shown are the growth curves of A549 cells expressing control, USP2-AS1, G3BP1 shRNA, or both USP2-AS1 and G3BP1 shRNA. Data shown are mean ± SD (n=3). **, $p < 0.01$; n.s., no significance.

(D) Colonies of A549 cells expressing control, USP2-AS1, G3BP1 shRNA, or both USP2-AS1 and G3BP1 shRNA were stained with crystal violet after 12 days of incubation. Data shown are mean ± SD (n=3). **, $p < 0.01$; ***, $p < 0.001$; n.s., no significance.

(E) Lysates from A549 cells expressing control, USP2-AS1, G3BP1 shRNA, or both

USP2-AS1 and G3BP1 shRNA were analyzed by western blotting.

(F) The relative expression levels of USP2-AS1 were examined by real-time RT-PCR for Fig. 6E. ***, $p < 0.001$; n.s., no significance.

(G) A549 cells were infected with lentiviruses expressing control, USP2-AS1 shRNA, G3BP1, or both USP2-AS1 shRNA and G3BP1 as indicated. Ninety-six hours later, cells were subjected to β-galactosidase staining. Data shown are mean ± SD (n=3). **, $p < 0.01$.

(H) Shown are the growth curves of A549 cells expressing control, USP2-AS1 shRNA, G3BP1, or both USP2-AS1 shRNA and G3BP1. Data shown are mean ± SD (n=3). **, $p < 0.01$.

(I) Colonies of A549 cells expressing control, USP2-AS1 shRNA, G3BP1, or both USP2-AS1 shRNA and G3BP1 were stained with crystal violet after 12 days of incubation. Data shown are mean ± SD (n=3). ***, $p < 0.001$.

(J) Lysates from A549 cells expressing control, USP2-AS1 shRNA, G3BP1, or both USP2-AS1 shRNA and G3BP1 were analyzed by western blotting.

(K) The relative expression levels of USP2-AS1 were examined by real-time RT-PCR for Fig. 6I. ***, $p < 0.001$; n.s., no significance.

(L and M) A549 cells were infected with lentiviruses expressing control shRNA, USP2-AS1 shRNA, USP2-AS1 shRNA plus shRNA-resistant USP2-AS1, or USP2-AS1 shRNA plus shRNA-resistant USP2-AS1 mutant (ΔG3BP1 BS) as indicated. (L) Ninety-six hours later, cells were subjected to β-galactosidase staining. (M) Shown are the relative expression levels of USP2-AS1 in each condition. Data shown are mean ± SD (n=3). **, $p < 0.01$; n.s., no significance.

(N) Shown are the growth curves of A549 cells expressing control, USP2-AS1, or USP2-AS1 mutant (ΔG3BP1 BS). Data shown are mean ± SD (n=3). **, $p < 0.01$; n.s., no significance.

(O) Colonies of A549 cells expressing control, USP2-AS1, or USP2-AS1 mutant (ΔG3BP1 BS) were stained with crystal violet after 12 days of incubation. Data shown are mean ± SD (n=3). ***, $p < 0.001$; n.s., no significance.

Figure S8. Related to Figure 7

(A) The relative expression levels of USP2-AS1 were examined by real-time RT-PCR for Fig. 7A. **, $p < 0.01$; ***, $p < 0.001$; n.s., no significance.

(B) The relative expression levels of USP2-AS1 were examined by real-time RT-PCR for Fig. 7C. **, $p < 0.01$; ***, $p < 0.001$; n.s., no significance.

(C) HCT116 cells expressing control, c-Myc, USP2-AS1 shRNA, or both c-Myc and USP2-AS1 shRNA were subjected to EdU incorporation assay. The shown images are representative of three independent experiments. Data shown are mean ± SD (n=3). **, $p < 0.01$; n.s., no significance.

(D) RNA from the excised xenografts (Fig. 7H) was analyzed by real-time RT-PCR to examine relative USP2-AS1 levels.

(E) Lysates from HCT116 and A549 cells expressing control shRNA, USP2-AS1 shRNA#1, or USP2-AS1 shRNA#2 were analyzed by western blotting.

(F) Lysates from HCT116 and A549 cells expressing control or USP2-AS1 were analyzed by western blotting.

Figure S1

A

NO.	transcript_id	log2(FC)	c-Myc CHIP-seq	
			A549	MCF7
1	ENST00000498979.6 (USP2-AS1)	3.86	√	√
2	ENST00000467458.2	3.07	√	√
3	ENST00000559041.1	2.48	√	√
4	ENST00000622757.1	2.36	√	√
5	ENST00000609153.1	2.13	√	√
6	ENST00000505731.5	1.88	√	√

B

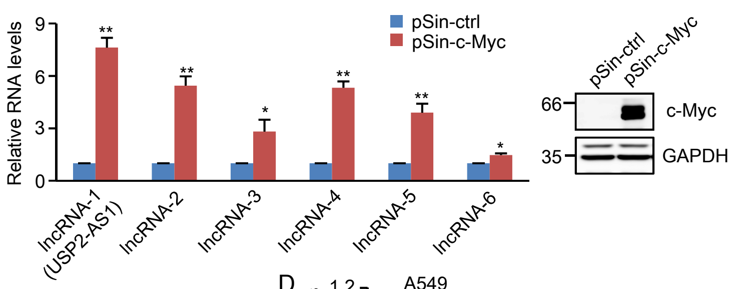

C

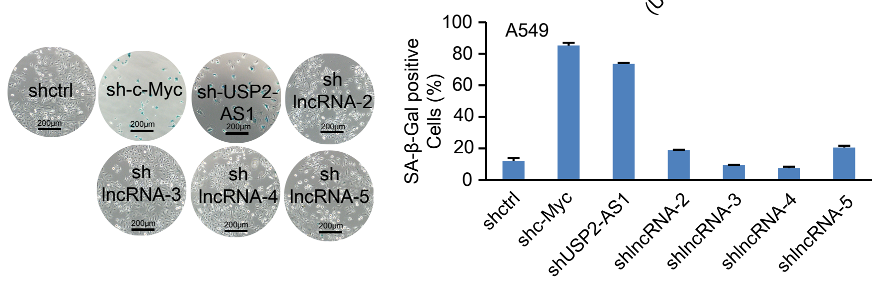

D

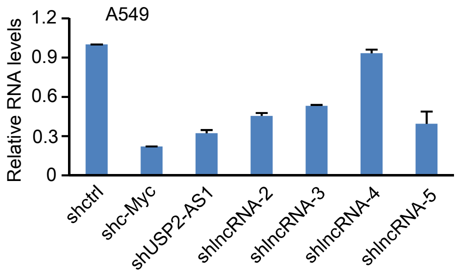

E

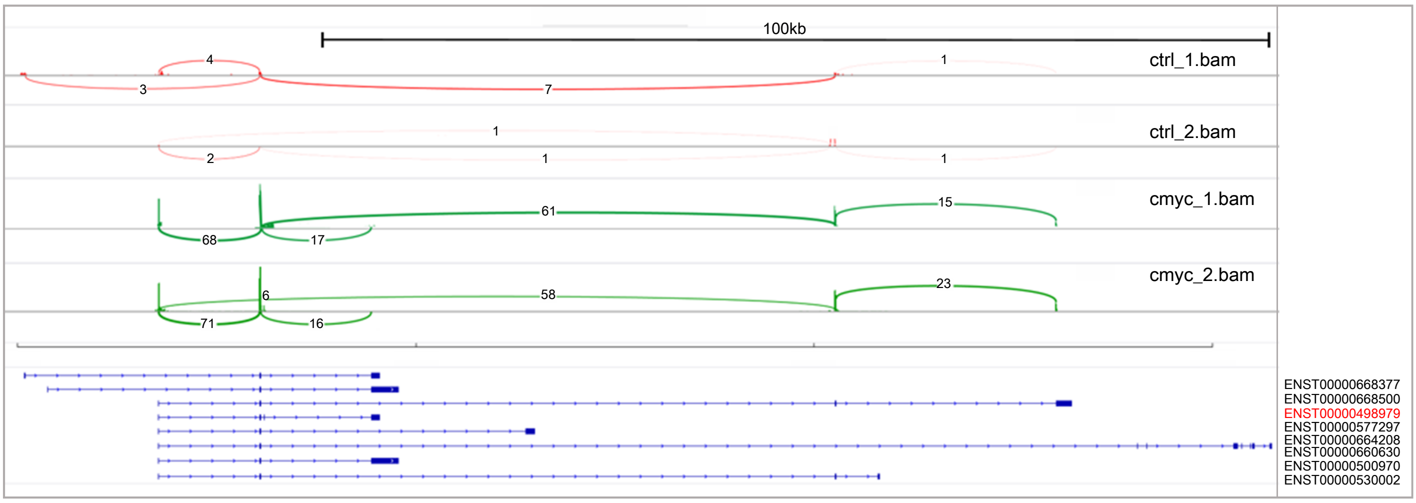

F

	ctrl_1	ctrl_2	c-myc_1	c-myc_2
ENST00000668377	0.051608	0.104922	0.104955	0.024161
ENST00000668500	0.051703	0	0	0.032904
ENST00000498979	0.121479	0.161841	1.928678	1.949841
ENST00000577297	0	0	0.159328	0.105523
ENST00000664208	0.021592	0.030416	0.376617	0.241002
ENST00000660630	0	0.024796	0.590782	0.622053
ENST00000500970	0.009562	0	0.269362	0.242487
ENST00000530002	0	0.046673	0.252159	0.304664

G H

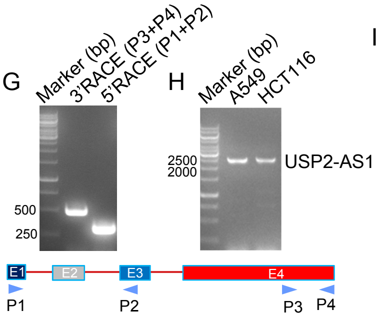

I

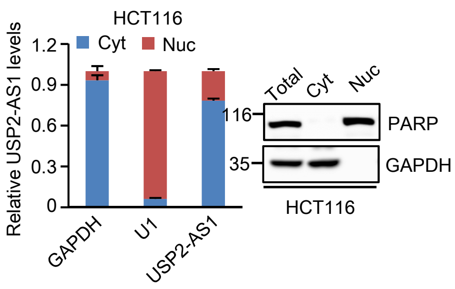

J

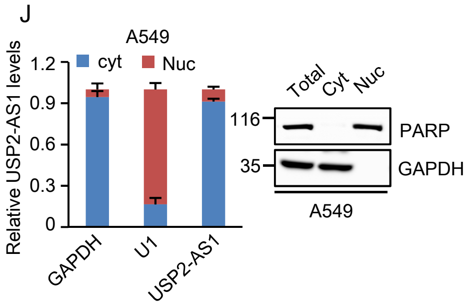

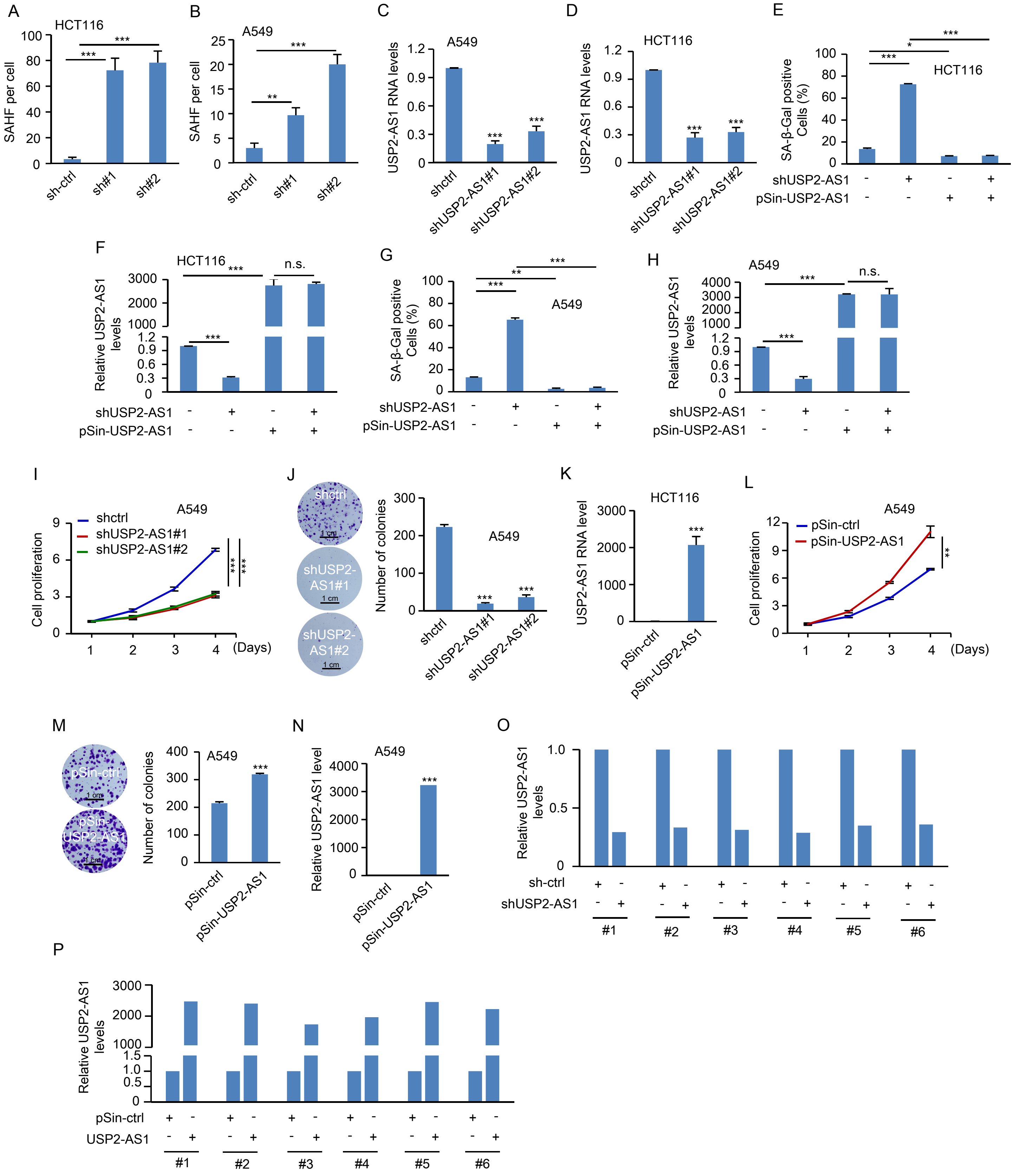
A
HCT116
SAHF per cell
sh-ctrl
sh#1
sh#2
B
A549
C
USP2-AS1 RNA levels
shctrl
shUSP2-AS1#1
shUSP2-AS1#2
D
E
SA-β-Gal positive Cells (%)
shUSP2-AS1
pSin-USP2-AS1
F
Relative USP2-AS1 levels
n.s.
G
H
I
Cell proliferation
(Days)
J
Number of colonies
1 cm
K
USP2-AS1 RNA level
pSin-ctrl
pSin-USP2-AS1
L
M
N
Relative USP2-AS1 level
O
sh-ctrl
#1
#2
#3
#4
#5
#6
P
pSin-ctrl
USP2-AS1

Figure S3

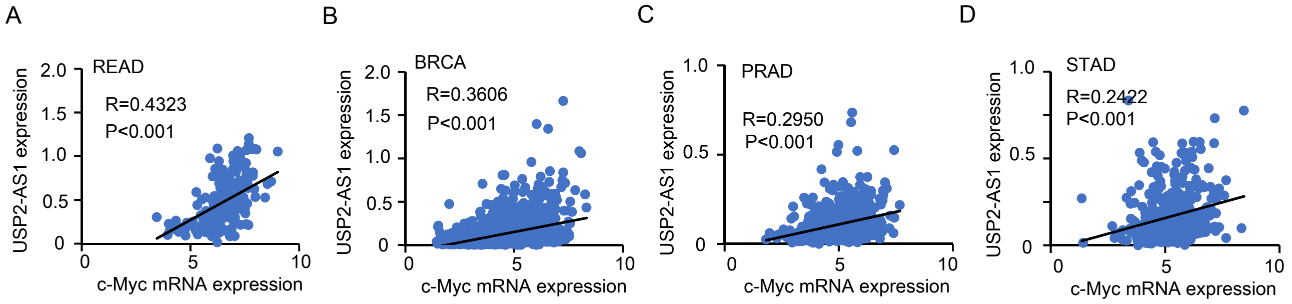

A
READ
R=0.4323
P<0.001
USP2-AS1 expression
c-Myc mRNA expression
B
BRCA
R=0.3606
P<0.001
USP2-AS1 expression
c-Myc mRNA expression
C
PRAD
R=0.2950
P<0.001
USP2-AS1 expression
c-Myc mRNA expression
D
STAD
R=0.2422
P<0.001
USP2-AS1 expression
c-Myc mRNA expression

Figure S4

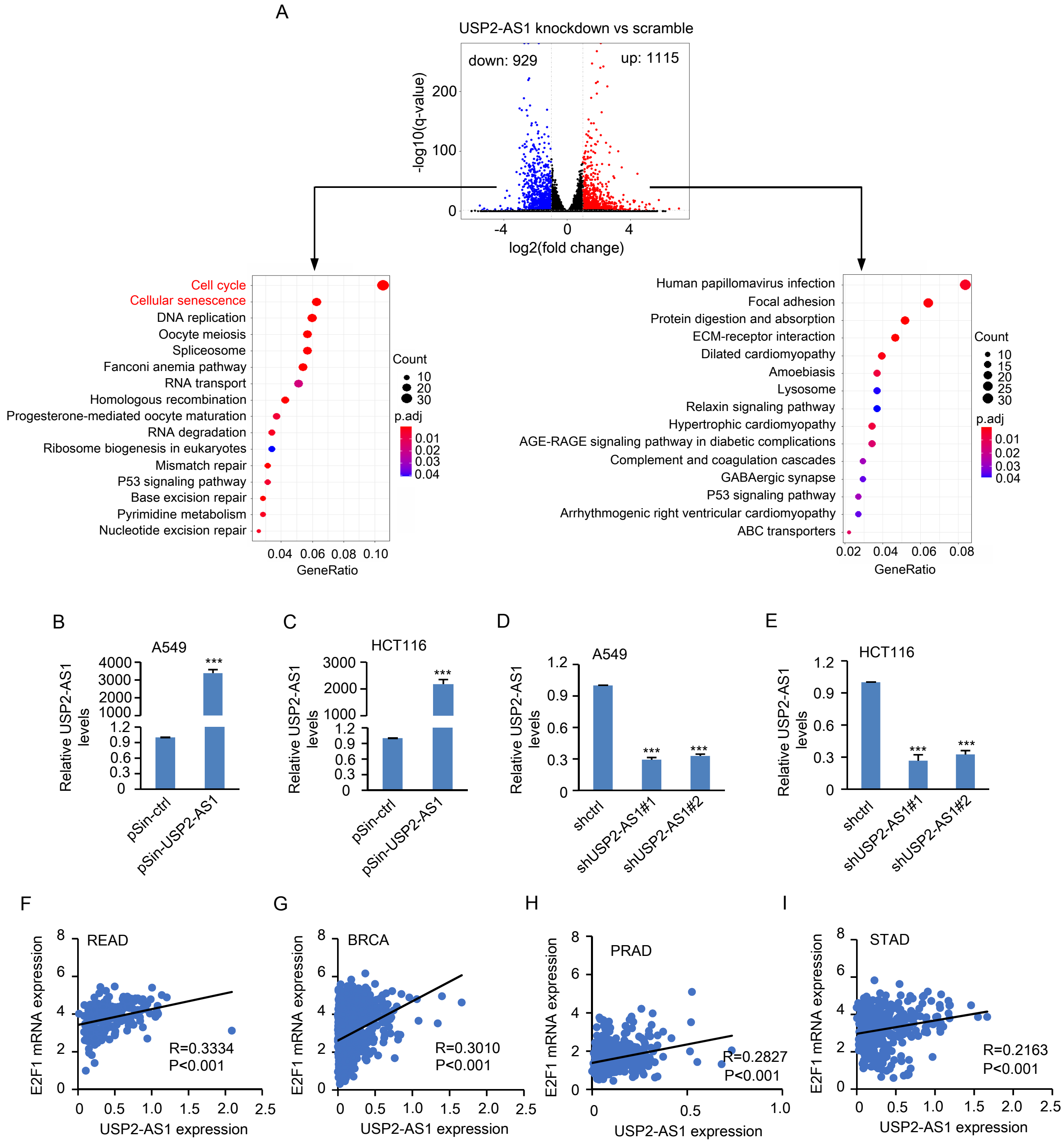

A
USP2-AS1 knockdown vs scramble
down: 929
up: 1115
-log10(q-value)
log2(fold change)
Cell cycle
Cellular senescence
DNA replication
Oocyte meiosis
Spliceosome
Fanconi anemia pathway
RNA transport
Homologous recombination
Progesterone-mediated oocyte maturation
RNA degradation
Ribosome biogenesis in eukaryotes
Mismatch repair
P53 signaling pathway
Base excision repair
Pyrimidine metabolism
Nucleotide excision repair
Count
p.adj
GeneRatio
Human papillomavirus infection
Focal adhesion
Protein digestion and absorption
ECM-receptor interaction
Dilated cardiomyopathy
Amoebiasis
Lysosome
Relaxin signaling pathway
Hypertrophic cardiomyopathy
AGE-RAGE signaling pathway in diabetic complications
Complement and coagulation cascades
GABAergic synapse
P53 signaling pathway
Arrhythmogenic right ventricular cardiomyopathy
ABC transporters
B
A549
Relative USP2-AS1 levels
pSin-ctrl
pSin-USP2-AS1
C
HCT116
D
A549
shctrl
shUSP2-AS1#1
shUSP2-AS1#2
E
HCT116
F
READ
R=0.3334
P<0.001
G
BRCA
R=0.3010
P<0.001
H
PRAD
R=0.2827
P<0.001
I
STAD
R=0.2163
P<0.001
E2F1 mRNA expression
USP2-AS1 expression

Figure S5

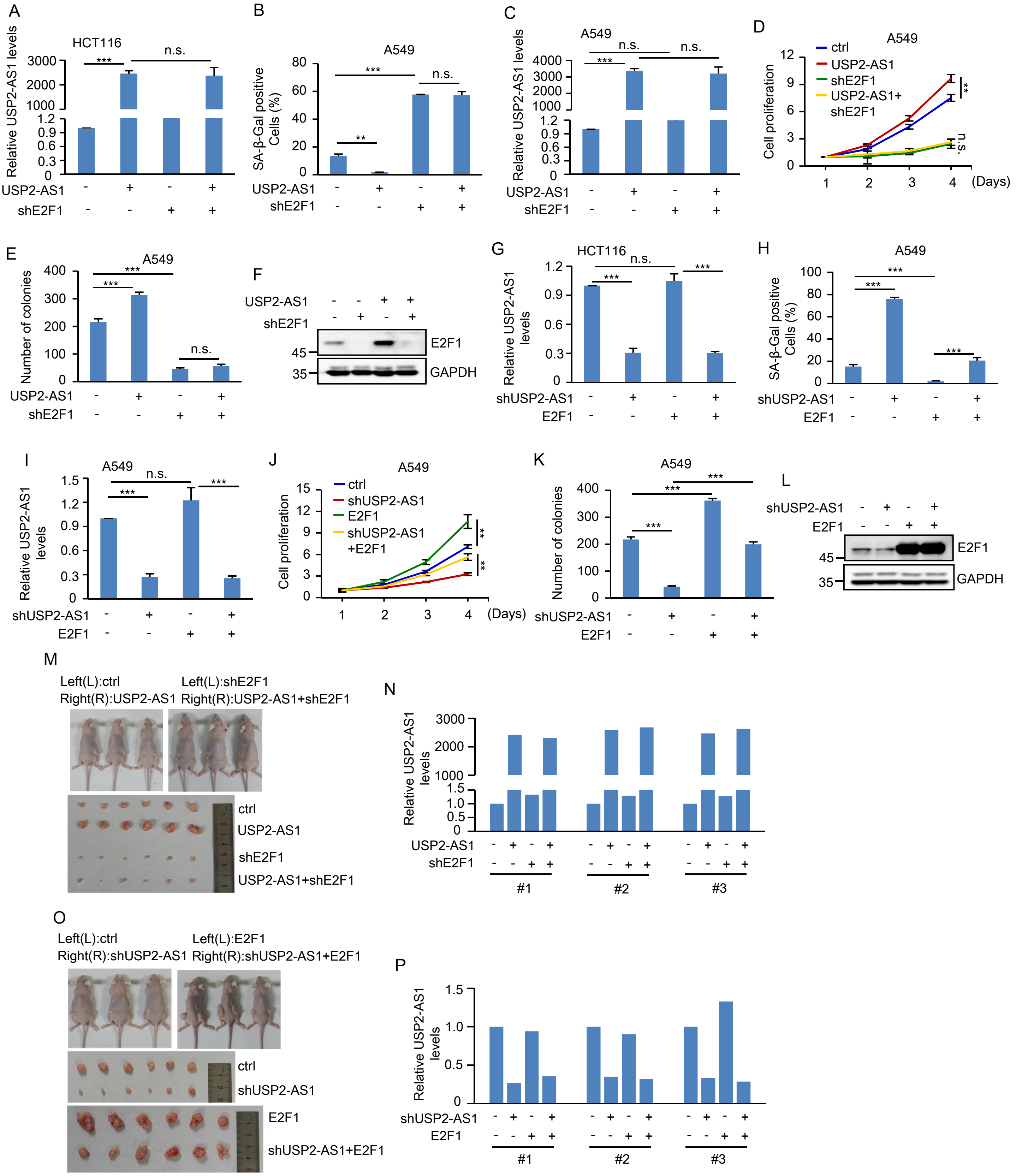

A
HCT116
Relative USP2-AS1 levels
USP2-AS1
shE2F1
n.s.
B
A549
SA-β-Gal positive Cells (%)
USP2-AS1
shE2F1
n.s.
C
A549
Relative USP2-AS1 levels
USP2-AS1
shE2F1
n.s.
D
A549
ctrl
USP2-AS1
shE2F1
USP2-AS1+ shE2F1
Cell proliferation
(Days)
n.s.
E
A549
Number of colonies
USP2-AS1
shE2F1
n.s.
F
USP2-AS1
shE2F1
E2F1
GAPDH
G
HCT116
Relative USP2-AS1 levels
shUSP2-AS1
E2F1
n.s.
H
A549
SA-β-Gal positive Cells (%)
shUSP2-AS1
E2F1
I
A549
Relative USP2-AS1 levels
shUSP2-AS1
E2F1
n.s.
J
A549
ctrl
shUSP2-AS1
E2F1
shUSP2-AS1 +E2F1
Cell proliferation
(Days)
K
A549
Number of colonies
shUSP2-AS1
E2F1
L
shUSP2-AS1
E2F1
E2F1
GAPDH
M
Left(L):ctrl
Right(R):USP2-AS1
Left(L):shE2F1
Right(R):USP2-AS1+shE2F1
ctrl
USP2-AS1
shE2F1
USP2-AS1+shE2F1
N
Relative USP2-AS1 levels
USP2-AS1
shE2F1
#1
#2
#3
O
Left(L):ctrl
Right(R):shUSP2-AS1
Left(L):E2F1
Right(R):shUSP2-AS1+E2F1
ctrl
shUSP2-AS1
E2F1
shUSP2-AS1+E2F1
P
Relative USP2-AS1 levels
shUSP2-AS1
E2F1
#1
#2
#3

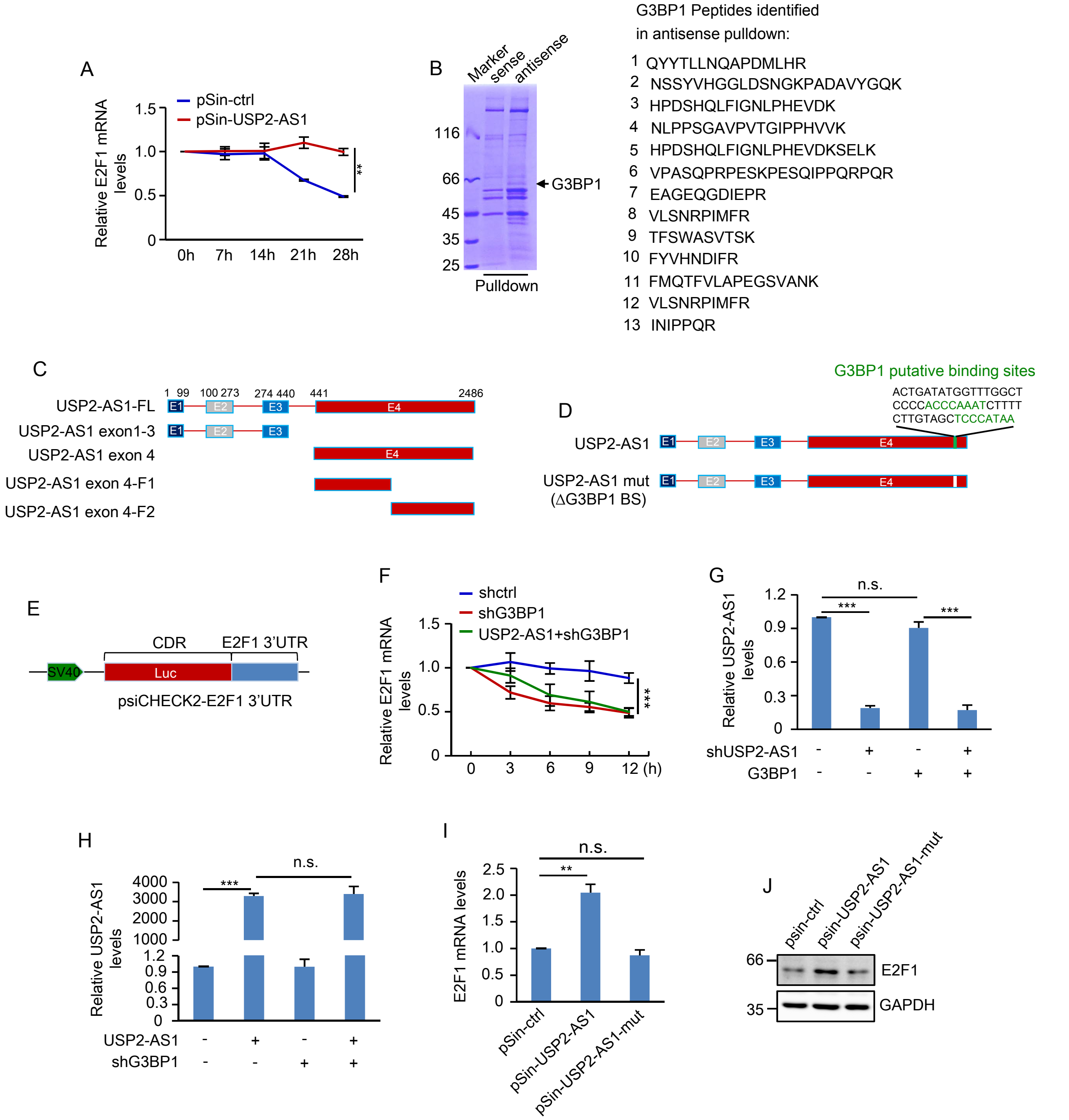
A
Relative E2F1 mRNA levels
pSin-ctrl
pSin-USP2-AS1
1.5
1.0
0.5
0
0h 7h 14h 21h 28h
**
B
Marker
sense
antisense
116
66
45
35
25
G3BP1
Pulldown
G3BP1 Peptides identified
in antisense pulldown:
1 QYYTLLNQAPDMLHR
2 NSSYVHGGLDSNGKPADAVYGQK
3 HPDSHQLFIGNLPHEVDK
4 NLPPSGAVPVTGIPPHVVK
5 HPDSHQLFIGNLPHEVDKSELK
6 VPASQPRPESKPESQIPPQRPQR
7 EAGEQGDIEPR
8 VLSNRPIMFR
9 TFSWASVTSK
10 FYVHNDIFR
11 FMQTFVLAPEGSVANK
12 VLSNRPIMFR
13 INIPPQR
C
1 99 100 273 274 440 441 2486
USP2-AS1-FL
USP2-AS1 exon1-3
USP2-AS1 exon 4
USP2-AS1 exon 4-F1
USP2-AS1 exon 4-F2
E1 E2 E3 E4
D
G3BP1 putative binding sites
ACTGATATGGTTTGGCT
CCCCACCCAAATCTTTT
CTTGTAGCTCCCATAA
USP2-AS1
USP2-AS1 mut
(ΔG3BP1 BS)
E
SV40
Luc
CDR
E2F1 3'UTR
psiCHECK2-E2F1 3'UTR
F
Relative E2F1 mRNA levels
shctrl
shG3BP1
USP2-AS1+shG3BP1
0 3 6 9 12 (h)
***
G
Relative USP2-AS1 levels
n.s.
***
shUSP2-AS1 - + - +
G3BP1 - - + +
H
Relative USP2-AS1 levels
4000
3000
2000
1000
1.2
0.9
0.6
0.3
0
***
n.s.
USP2-AS1 - + - +
shG3BP1 - - + +
I
E2F1 mRNA levels
2.5
2.0
1.5
1.0
0.5
0
n.s.
**
pSin-ctrl
pSin-USP2-AS1
pSin-USP2-AS1-mut
J
psin-ctrl
psin-USP2-AS1
psin-USP2-AS1-mut
66
35
E2F1
GAPDH

Figure S7

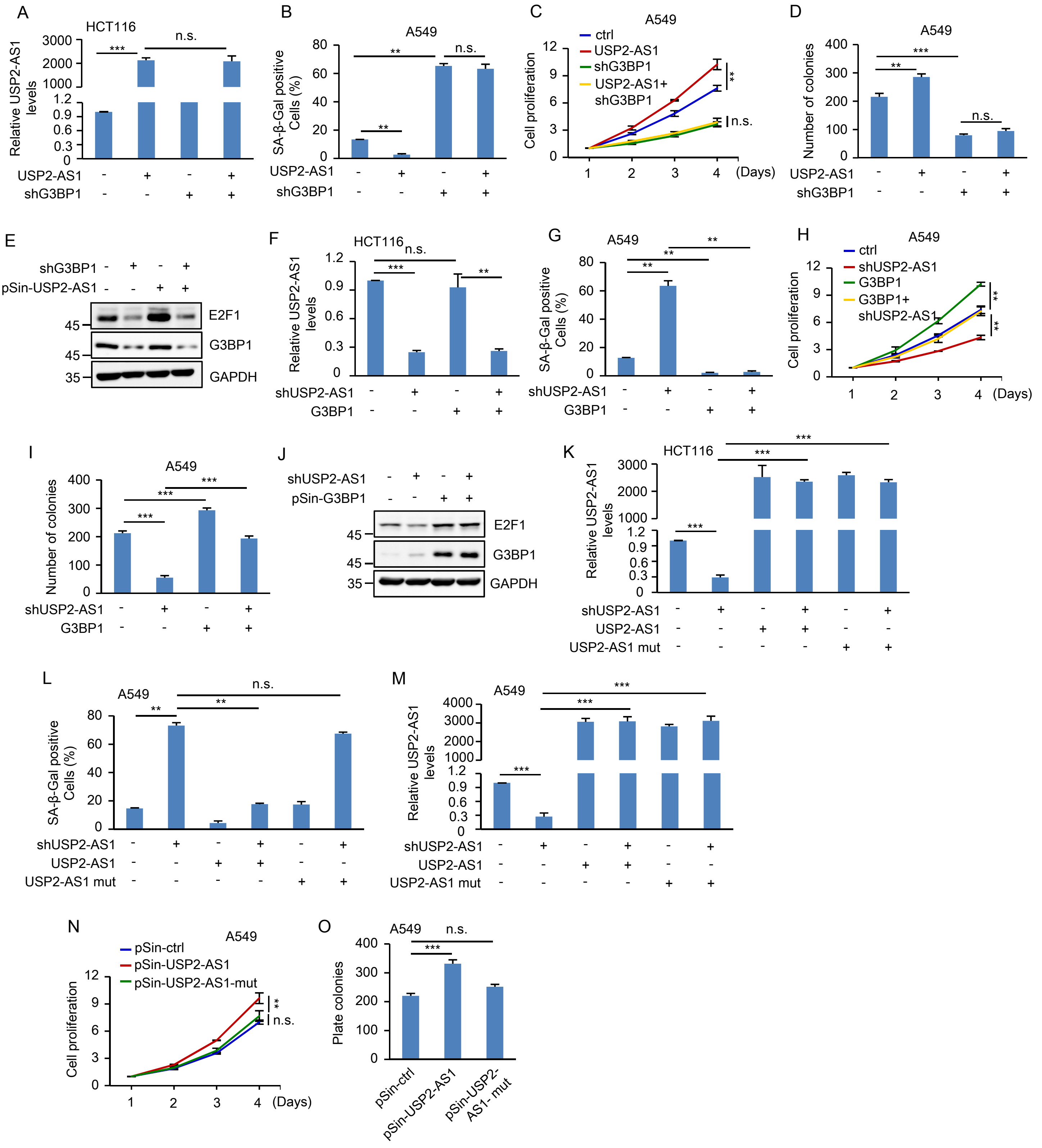
A
HCT116
Relative USP2-AS1 levels
***
n.s.
USP2-AS1
shG3BP1
B
A549
SA-β-Gal positive Cells (%)
**
n.s.
USP2-AS1
shG3BP1
C
A549
ctrl
USP2-AS1
shG3BP1
USP2-AS1+ shG3BP1
Cell proliferation
(Days)
n.s.
D
A549
Number of colonies
USP2-AS1
shG3BP1
E
shG3BP1
pSin-USP2-AS1
E2F1
G3BP1
GAPDH
F
HCT116
Relative USP2-AS1 levels
shUSP2-AS1
G3BP1
G
A549
SA-β-Gal positive Cells (%)
shUSP2-AS1
G3BP1
H
A549
ctrl
shUSP2-AS1
G3BP1
G3BP1+ shUSP2-AS1
Cell proliferation
(Days)
I
A549
Number of colonies
shUSP2-AS1
G3BP1
J
shUSP2-AS1
pSin-G3BP1
E2F1
G3BP1
GAPDH
K
HCT116
Relative USP2-AS1 levels
shUSP2-AS1
USP2-AS1
USP2-AS1 mut
L
A549
SA-β-Gal positive Cells (%)
shUSP2-AS1
USP2-AS1
USP2-AS1 mut
M
A549
Relative USP2-AS1 levels
shUSP2-AS1
USP2-AS1
USP2-AS1 mut
N
A549
pSin-ctrl
pSin-USP2-AS1
pSin-USP2-AS1-mut
Cell proliferation
(Days)
O
A549
Plate colonies
pSin-ctrl
pSin-USP2-AS1
pSin-USP2-AS1- mut

Figure S8

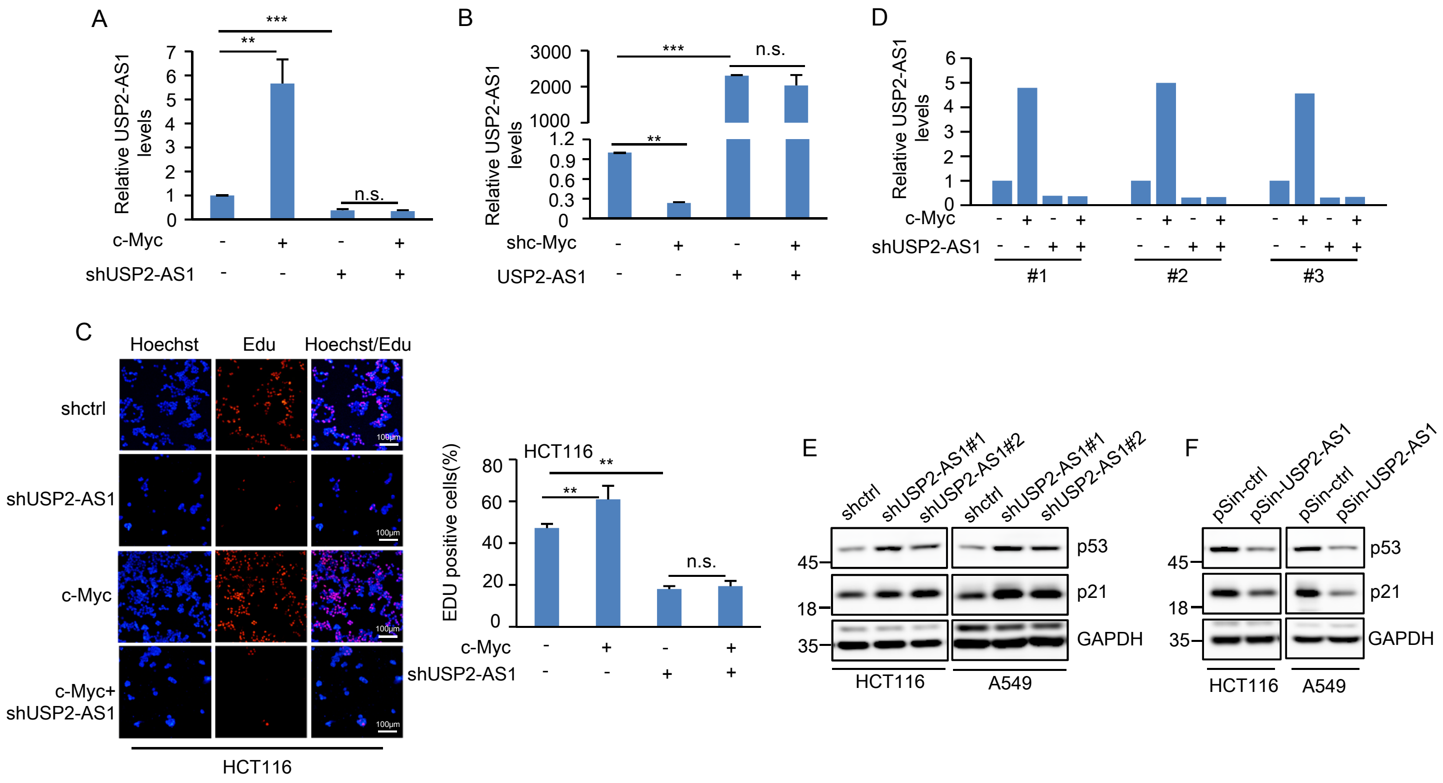

A
Relative USP2-AS1 levels
c-Myc
shUSP2-AS1
n.s.
B
Relative USP2-AS1 levels
shc-Myc
USP2-AS1
n.s.
D
Relative USP2-AS1 levels
c-Myc
shUSP2-AS1
#1
#2
#3
C
Hoechst
Edu
Hoechst/Edu
shctrl
shUSP2-AS1
c-Myc
c-Myc+ shUSP2-AS1
HCT116
EDU positive cells(%)
E
shctrl
shUSP2-AS1#1
shUSP2-AS1#2
p53
p21
GAPDH
A549
F
pSin-ctrl
pSin-USP2-AS1
